# Evaluation of resource and environmental carrying capacity in rare earth mining areas in China

**DOI:** 10.1038/s41598-022-10105-2

**Published:** 2022-04-12

**Authors:** Jingjing Bai, Xin Xu, Yaoting Duan, Guangyu Zhang, Zhe Wang, Lu Wang, Chunli Zheng

**Affiliations:** 1 School of Energy and Environment, Inner Mongolia University of Science and Technology, Baotou, 014010 Inner Mongolia China; 2 Engineering Research Center of Evaluation and Restoration in the Mining Ecological Environments, Inner Mongolia University of Science & Technology, Baotou, 014010 Inner Mongolia China; 3 Ganjiang Innovation Academy, Chinese Academy of Sciences, Ganzhou, 341000 Jiangxi China

**Keywords:** Ecology, Environmental sciences, Environmental social sciences

## Abstract

Rare earth elements are a nonrenewable and important strategic resource, and China is rich in these elements. However, the substantial exploitation of these resources has caused the migration, diffusion, transformation and accumulation of pollution sources, which in turn has a profound impact on the ecological environment of mining areas. Accurate evaluations of resource and environmental carrying capacity (RECC) are important for the green development of mining areas. In this paper, the fuzzy comprehensive evaluation method based on the combination of the AHP (Analytic Hierarchy Process) and entropy methods is used to study the RECC of mine areas in terms of both support capacity and pressure. The Bayan Obo mine in Inner Mongolia, the Longnan mine in Jiangxi, the Weishan mine in Shandong, the Mianning mine in Sichuan, the Pingyuan mine in Guangdong, and the Chongzuo mine in Guangxi, which are typical representative mines, were selected for a horizontal comparison. The results show that, with the exception of the Bayan Obo mine, the support index was greater than the pressure index in terms of mining and human activities in all mining areas. The RECC index ranked order for the mining areas was Bayan Obo > Longnan > Mianning > Pingyuan > Weishan > Chongzuo. In addition, an obstacle degree model was used to identify and extract the main factors affecting the ecological quality of the mine sites. The ratio of investment in environmental pollution control to GDP was the most important factor, of all factors, which limited the improvement in the mine support index. Through the above research, we identified the main factors affecting the ecological carrying capacity of each mining area, providing a scientific basis for formulating corresponding environmental regulations and reducing the environmental pollution caused by rare earth mining.

## Introduction

### Background

Rare earth elements are key elements that are indispensable to the transformation of traditional industries, the development of new industries and the national defense science and technology industry, as they are a nonrenewable and important strategic resource^1^. Rare earth functional elements are used in new energy vehicles, national defense equipment, rare earth permanent magnet motors, energy savings and environmental protection efforts, rail transportation, new materials, new energy products and other key areas of vigorous development^2–6^ and these uses drive the demand for the synergistic growth of rare earth products and promote the steady development of the rare earth industry. The strategic and economic value of rare earth elements are becoming increasingly prominent.

**Figure 1:**
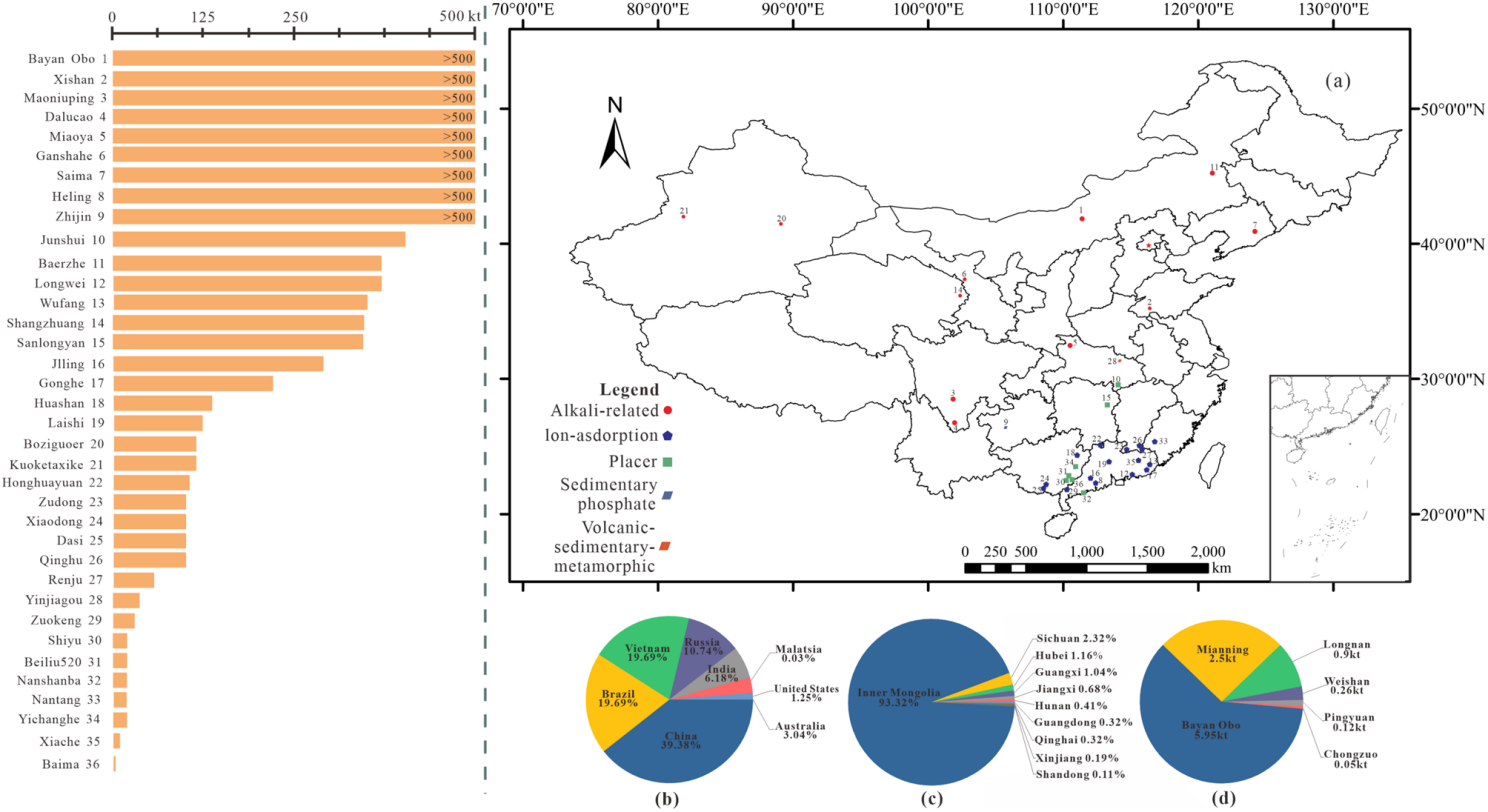
Distribution of rare earth mines in China. (**a**) 36 rare earth mine types and reserves; (**b**) reserves of rare earths in major countries in the world; (**c**) reserves of rare earths in major provinces in China; (**d**) annual mining capacity of rare earths in major mining areas in China.

Global rare earth element reserves are abundant and widely distributed in 38 countries on all continents, with identified resource reserves of over 200 million tons^7^. With the discovery and exploitation of new rare earth elements in every country, the pattern of rare earth element reserves in the world is changing. China has abundant rare earth elements, with all types of deposits and rare earth elements distributed in 22 provinces (Fig. 1). These resources are widely distributed and relatively concentrated accounting for 30% of the world's total, as the world's largest reserves, with China supplying more than 58% of the world's rare earth element market^8^. Thus, China has provided the world with an excessive amount of rare earth products^9^. Since rare earth elements are widely scattered on the Earth's crust, mining is difficult and extremely expensive, and the process of resource development has a negative impact on local water bodies, the atmosphere, soil, organisms, and other environmental elements that are closely related to human survival. Mining wastewater from rare earth production can acidify the surrounding soil and groundwater. Mining solid waste can produce radioactive materials and heavy metal contamination^10–12^. Currently, mining is the most important activity destroying the ecological environment and causing pollution and disasters. The development of rare earth elements, due to their unique form of extraction and metallurgy, has caused substantial damage to local ecological environments.

Cross-sectional comparisons among rare earth element mining areas help fill the gaps in understanding the RECC in rare earth element mining areas and clarify the developmental differences among rare earth element mining areas in terms of their ecology, environments, and economies. Furthermore, conducting dynamic change analysis helps capture the developmental fluctuations in each mining area across a time scale. This paper selects the following typical mines as representative research objects: the Inner Mongolia Bayan Obo mine, Jiangxi Longnan mine, Shandong Weishan mine, Sichuan Mianning mine, Guangdong Pingyuan mine and Guangxi Chongzuo mine. These mining areas account for more than 90% of the total rare earth elements mined in China, so they are considered representative of the mines in China. The results of this study can be used to formulate strategic policies according to local conditions.

### Literature review

The carrying capacity is defined as the ability of the carrier to support the "carrying object"^13^. Hadwen and Palmer^14^ considered carrying capacity to be the number of lives that can be sustained without destroying the ecosystem, emphasizing the goal of not destroying the ecosystem to achieve sustainable development. In the 1970s, Holling^15^ introduced the concept of RECC as the ability of an ecosystem to resist external disturbances and maintain the relative stability of its original ecological structure. With the development of economies and societies, people have gradually paid attention to the impact of human activities on the ecological environment. Chapman et al.^16^ noted that RECC overload has become a common problem in countries around the world.

In fact, RECC is an evolutionary process because it is influenced by various dynamic factors, such as human activities, energy structure and consumption, and climate change. Therefore, from an evolutionary point of view, it is necessary and appropriate to examine the RECC over time. However, unlike physical objects, ecosystems are not static but dynamic and variable^17^. Thresholds for RECC do not actually occur because of the inability to conduct anthropogenic system destruction experiments^18^.

Carrying capacity research has mostly been carried out regionally, and due to the variability in each region, most of the research first analyzed the type of carrying capacity, determined the indicators affecting the carrying capacity, established an evaluation index system, determined the index weights, and then completed the evaluation through various evaluation methods^19–21^. In the evaluation process, a researcher determines the results of this type of study in two ways: the first process assumes that resource, environmental, economic, social and other criteria are additive through the positive and negative characteristics of the indicators^21–23^; the other process is to construct a pressure-state-response as a system layer^18,19^. Most studies divide the RECC system into ecological, environmental and human-social criteria and calculate the indices of each criterion layer as a whole. However, this division ignores the supporting capacity of the resource environment and the overall pressure of human activities on these systems and is not conducive to further understanding the supporting activities of a resource environment and the impact of human activities on these systems. The attribute characteristics of these two relatively independent systems are not portrayed to facilitate an understanding of the current status of these two systems^19^.

## Research methodology and data sources

### Theoretical content and construction of the RECC indicator system

Ideally, an ecosystem maintains its functions in a relatively dynamic state of equilibrium^18^. However, as human activities increase the load on the resource environment or degrade the supporting capacity, this balance breakdown will occur when load pressure > supporting capacity. This study integrates the interaction between carriers and loads by examining the relationship between RECC ecosystem loads and ecosystem carriers.

Through the analysis of numerous factors affecting the ecological carrying capacity, we obtained a final system of carrying capacity indicators. In this study, the carrying capacity was divided into two aspects: the support surface was divided into four intermediate layers of climatic conditions, resource endowment, environmental management and economic development, and the pressure surface was divided into three intermediate layers of ecological damage loss, environmental pollution loss and social pressure. The support system included both climatic conditions and resources. The resources included both natural resources and social resources. Social resources mainly refer to environmental governance and good economic development formed through human capital, production and technology, such as the comprehensive utilization rate of industrial solid waste, urban sewage treatment rate, and harmless treatment rate of domestic waste. The pressure of the RECC system included ecological damage and environmental pollution losses, which originate from human activities. Human activities mainly included two parts: social activity, such as economic growth, social development, entertainment and personal enjoyment, and social production, such as mining and smelting.

The evaluation indictor system for the RECC of rare earth element mining areas incorporated 30 indicators. Among them, the 18 indicators shown in Table 1 were basic indicators for the development of mining areas, common indicators in previous studies on ecological carrying capacity, and 12 new indicators, S_2-4_, S_2-5_, S_4-3_, P_1-1_, P_1-2_, P_1-3_, P_1-4_, P_2-1_, P_2-2_, P_2-3_, P_2-4_, and P_3-6_, representing the characteristic indicators of rare earth element mining areas. Sixteen support indicators and 14 pressure indicators were identified by combining the data available for the 6 mining areas (Table 2). In the selection of indicators, the percentage category and per capita category were selected to maintain consistency in the evaluation. For certain indicators describing local characteristics, such as GDP per capita and share of secondary industry in GDP, county-level administrative district data were specifically selected because in comparison to provinces and cities, the most basic government unit in China, the county (district), can better capture the heterogeneity in mining areas.

**Table 1:** Summary of typical ecological base evaluation indicators in previous studies.

Indicators (units)		References
S_1-1_	Frost free period (days)	^24^
S_1-2_	Annual average relative humidity (%)	^25^
S_1-3_	Annual average temperature (℃)	^24,25^
S_2-1_	Total annual precipitation (mm)	^26^
S_2-2_	Arable land to regional area (%)	^23,27–30^
S_2-3_	Forest-grassland coverage (%)	^23,27,31^
S_2-6_	Water resources per capita (m^3^)	^29,30,42^
S_3-1_	Comprehensive utilization rate of industrial solid waste (%)	^30,32,33^
S_3-2_	Urban sewage treatment rate (%)	^20^
S_3-3_	Harmless treatment rate of domestic waste (%)	^23^
S_3-4_	Environmental pollution control investment to GDP ratio (%)	^20^
S_4-1_	Foreign exchange earnings from tourism (USD million)	^26^
S_4-2_	GDP per capita (RMB)	^27–30^
P_3-1_	Urban registered unemployment rate (%)	^23^
P_3-2_	Share of secondary industry in GDP (%)	^23,29,30,34^
P_3-3_	Urban per capita daily domestic water consumption (L)	^20^
P_3-4_	Natural population growth rate (%)	^23,27^
P_3-5_	Energy consumption of 10,000 Yuan GDP (t standard coal)	^23,30,32^

**Table 2:** Evaluation index system of the RECC.

System	Criteria layer	Indicators (units)		System	Criteria layer	Indicators (units)	
Support	Climate Conditions	S_1-1_	Frost free period (days)	Pressure	Ecological damage loss	P_1-1_	Loss of ecological value volume of organic matter due to rare earth mining Ten thousand yuan)
		S_1-2_	Annual average relative humidity (%)			P_1-2_	Rare earth mining leads to the loss of value quantity of released O^2^ and fixed CO^2^ (Ten thousand yuan)
		S_1-3_	Annual average temperature (℃)			P_1-3_	Rare earth mining leads to the loss of water conservation value amount (Ten thousand yuan)
	Resource Endowment	S_2-1_	Total annual precipitation (mm)			P_1-4_	Rare earth mining leads to the loss of soil conservation value amount (Ten thousand yuan)
		S_2-2_	Arable land to regional area (%)				
		S_2-3_	Forest-grassland coverage (%)		Environmental pollution loss	P_2-1_	Rare earth smelting water pollution treatment cost accounting (Ten thousand yuan)
		S_2-4_	Rare earth resources reserves (million tons)			P_2-2_	Rare earth smelting air pollution treatment cost accounting (Ten thousand yuan)
		S_2-5_	Rare earth resources reserves (million tons)			P_2-3_	Rare earth smelting solid waste pollution treatment cost accounting (Ten thousand yuan)
		S_2-6_	Water resources per capita (m^3^)			P_2-4_	The radioactivity (nGy/h)
	Environmental Governance	S_3-1_	Comprehensive utilization rate of industrial solid waste (%)				
		S_3-2_	Urban sewage treatment rate (%)		Social pressure	P_3-1_	Urban registered unemployment rate (%)
		S_3-3_	Harmless treatment rate of domestic waste (%)			P_3-2_	Share of secondary industry in GDP (%)
		S_3-4_	Environmental pollution control investment to GDP ratio (%)			P_3-3_	Urban per capita daily domestic water consumption (L)
	Economic Development	S_4-1_	Foreign exchange earnings from tourism (USD million)			P_3-4_	Natural population growth rate (%)
		S_4-2_	GDP per capita (RMB)			P_3-5_	Energy consumption of 10,000 Yuan GDP (t standard coal)
		S_4-3_	Number of Rare Earth Related Employees (Number)			P_3-6_	Annual mining volume (million tons)

The steps of the RECC calculation in this study were divided into (1) construction of a standardized evaluation matrix; (2) standard normalization of indicators; (3) determination of indicator weights using a combination of AHP and the entropy value method; and (4) use of the linear weighting method to obtain the support index, pressure index and RECC index. Since the support index and pressure index rely on the same weighting method, the support index is described as an example.

### Calculation of the RECC index

The indicators are normalized first, and the weight of each indicator is determined by the fuzzy comprehensive evaluation method (the details are provided in the Supplementary Materials). According to the normalized values of each indicator and the corresponding weights, the support index and pressure index can be obtained using the linear weighting method, as shown in Eqs. (1) and (2):

1 $${S}_{i}=\sum_{i=1}^{n}{S}_{ij}{W}_{i}^{s}$$

2 $${P}_{i}=\sum_{i=1}^{n}{P}_{ij}{W}_{i}^{p}$$

The RECC index for the study area was equal to the ratio of the stress index to the support index.

3 $${C}_{i}=\frac{{P}_{i}}{{S}_{i}}$$

### RECC level classification and coupling mechanism

#### Support surface and pressure surface evaluation grade classification

A hierarchical approach was used to classify RECC for more targeted information on human activities. This was previously shown to be an effective method for analyzing trends in results^21^.

**Figure 2:**
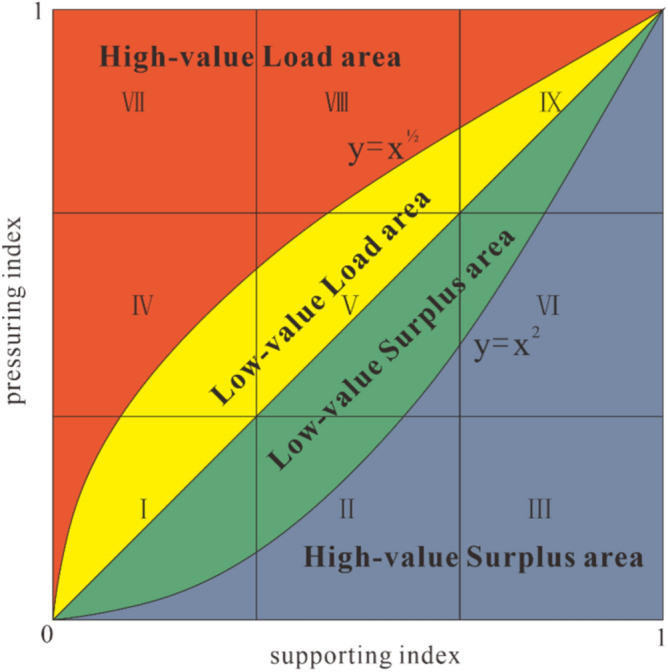
Schematic diagram of support index and pressure index zoned states and coupling curves.

First, the support and pressure surfaces were divided into low, medium, and high grades for measurements. The equal scoring method was used for grading, and this method was also applied in a previous study^20,35^. According to this approach, the three levels were equally distributed in the range of (0,1). The support index was set to S, and the pressure index was set to P (Table 3). A total of 9 blocks were divided into categories, I, II, III, IV, V, VI, VII, VIII, and IX, representing 9 different integrated support and pressure index conditions within the mine area, the details of which are shown in Table 3 and Fig. 2.

**Table 3:** Evaluation grade classification of support surface and pressure surface.

Evaluation grade division	Low level (L)	Medium level (M)	High level (H)
P	0 ≤ P ≤ 0.33	0.33 < P ≤ 0.66	0.66 < P ≤ 1
S	0 ≤ S ≤ 0.33	0.33 < S ≤ 0.66	0.66 < S ≤ 1

#### RECC coupled model

Theoretically, there were three results for the load capacity index, depending on the magnitude of the support and pressure indices, C < 1(P < S), C = 1(P = S), and C > 1(P > S); when C < 1(P < S); the pressures generated by human activities were less than the capacity of the resource-environment system to support them, such as for II, III, VI. When C = 1 (P = S), the supporting capacity of resource and environmental enrichment was generally equal to the pressure generated by human activities, such as for I, V and IX. When C > 1 (P > S), the pressure generated by human activities was greater than the capacity of the resource environment system to support it, and the RECC in the area was overloaded, such as for IV, VII and VIII.

We used $y={x}^{2}$ and $y=\sqrt{x}$ as criteria to classify the carrying capacity index into 4 regions^19,34^, as shown in Table 4 and Fig. 2.

**Table 4:** Significance of the partition of the coupling mechanism between the support index and the pressure index.

Partition state	$0\le \mathrm{y}\le {x}^{2}$	${x}^{2}\le \mathrm{y}<\mathrm{x}$	$\mathrm{x}\le \mathrm{y}<\sqrt{x}$	$\sqrt{x}\le \mathrm{y}\le 1$
$0 \le \mathrm{ x }\le 1$	High-value Surplus area	Low-value Surplus area	Low-value Load area	High-value Load area

### Obstacle degree model

On the basis of the carrying capacity indicator system, each indicator was further analyzed to determine the main etiological factors hindering the development of the study area, with reference to the existing literature^19,21^. The main purpose was to determine the obstacle factors using two indicators: indicator deviation degree and obstacle degree, and the basic mathematical formula is:

4 $${I}_{i}=1-{X}_{i}$$

5 $${A}_{i}=\frac{{I}_{i}{W}_{i}}{{\sum }_{j=1}^{m}{I}_{i}{W}_{i}}$$

where ${X}_{i}$ is the standardized value of a single indicator; ${I}_{i}$ is the difference between the evaluated value of a single indicator and 100%, i.e., the indicator deviation; ${W}_{i}$ is the weight of the ith single indicator; ${A}_{i}$ is the indicator barrier; and *m* is the number of evaluation indicators.

### Data sources

This study aimed to assess and compare the ecological and environmental carrying capacities of six typical rare earth element mining areas in China during 2012–2019. The raw data for most of the indicators listed in Table 1 were obtained from the 2012–2017 statistical yearbooks of the administrative regions where each study area is located and the China County Statistical Yearbook and Department of Rural Social and Economic Survey^36^, Baotou City Bureau of Statistics^37^, Jiangxi Bureau of Statistics^38^, Jining Bureau of Statistics^39^, Liangshan Yi Autonomous Prefecture People's Government^40^, Meizhou yearbook^41^, and Chongzuo local history compilation committee^42^. The original data on rare earth element reserves and the proportion of medium and heavy rare earth elements are from Rare Earth Mining and Environmental Protection^9^. Some of the data and calculation methods for ecological damage loss and environmental pollution loss indicators were obtained from the Ecological and Environmental Cost Assessment of Rare Earth Resource Development in China from 2001–2013^43^, and details of the methodology are described in Part 4 and Part 5 of the Supplementary Material. The original radioactivity data were obtained from research papers^44–48^, and data on rare earth element policies and the annual mining volume of rare earth elements were obtained from national database statistics (http://www.mnr.gov.cn/dt/ywbb/).

## Results

### Comprehensive weights of the evaluation indices by the AHP and entropy methods

**Figure 3:**
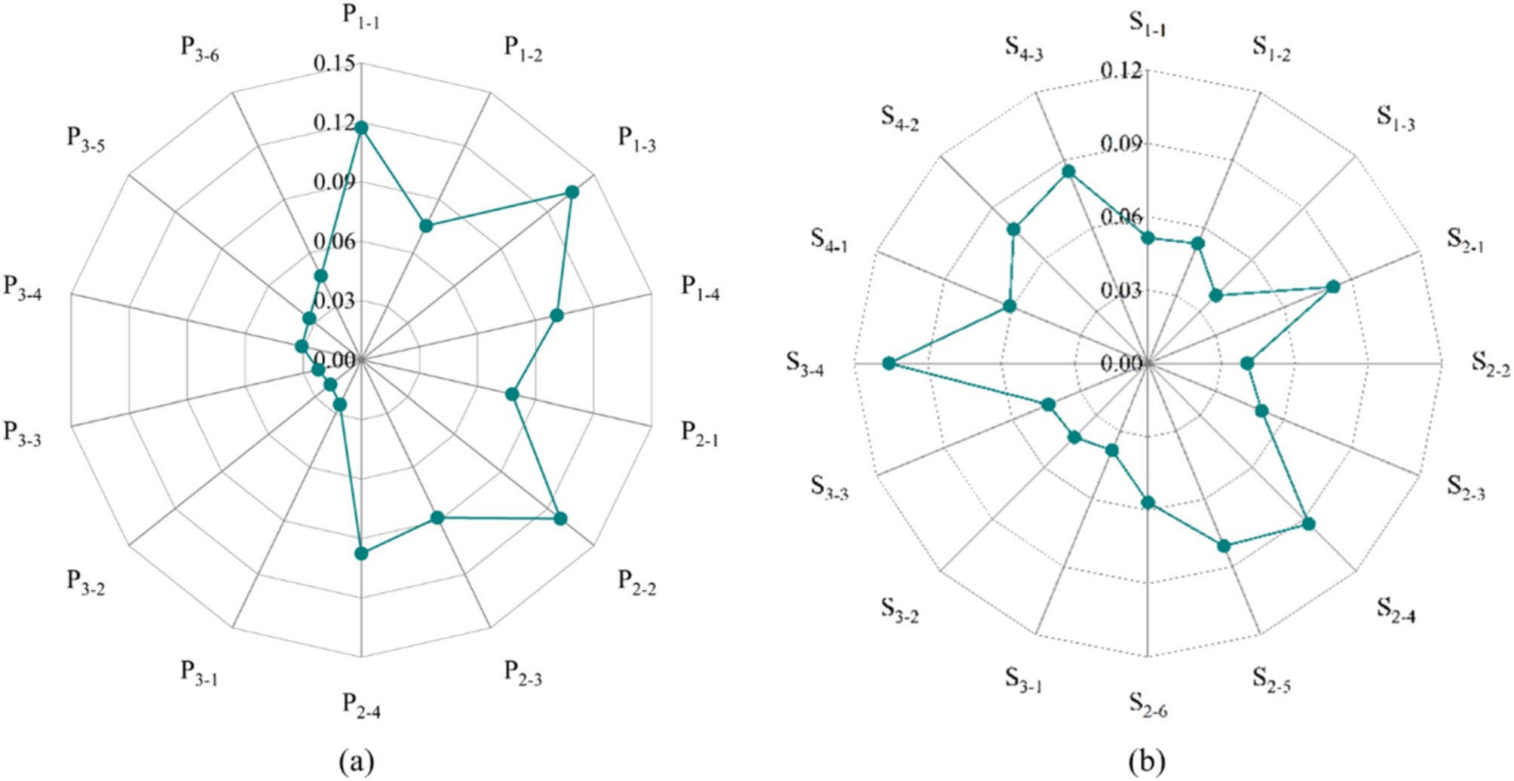
Comprehensive weights of evaluation indicators. (**a**) Pressure indicators weights; (**b**) Support indicators weights.

According to the construction of the evaluation indicator system, the weights of each indicator were first determined (Fig. 3). The indicators with greater weight in the pressure system were the following: loss of value and quantity of water due to rare earth element mining (0.1331) > the cost of air pollution control of rare earth element smelting (0.1255) > loss of ecological value and quantity of organic substances due to rare earth element mining (0.1145) > loss of value and quantity of soil conservation due to rare earth element mining ((0.0983) > radioactivity (0.0948). The loss of water value and quantity due to rare earth element mining, the loss of organic matter ecological value and quantity due to rare earth element mining, and the loss of soil conservation value and quantity due to rare earth element mining were indicators of ecological damage losses. The rare earth element smelting air pollution control cost and radioactivity were indicators of environmental pollution losses. The weights of each subsystem of the pressure system were 0.4183 (ecological damage loss), 0.3809 (environmental pollution loss), and 0.2008 (social pressure).

The indicators with higher weights in the support system were the following: investment in environmental pollution control to GDP ratio (0.1006) > rare earth element reserve (0.0877) > rare earth element policy (0.0809) > number of rare earth element-related employees (0.0799) > total annual precipitation (0.0767). The ratio of investment in environmental pollution control to GDP is an indicator of environmental control, the amount of rare earth element reserves is an indicator of resource endowment, the rare earth element policy and the number of rare earth element-related employees are indicators of economic development, and the total annual precipitation is an indicator of climate. The weights of the subsystems of the support system were 0.1282 (climatic conditions), 0.3723 (resource endowment), 0.2104 (environmental governance), and 0.2892 (economic development).

### Analysis of the RECC at the mining sites using a coupled model

**Figure 4:**
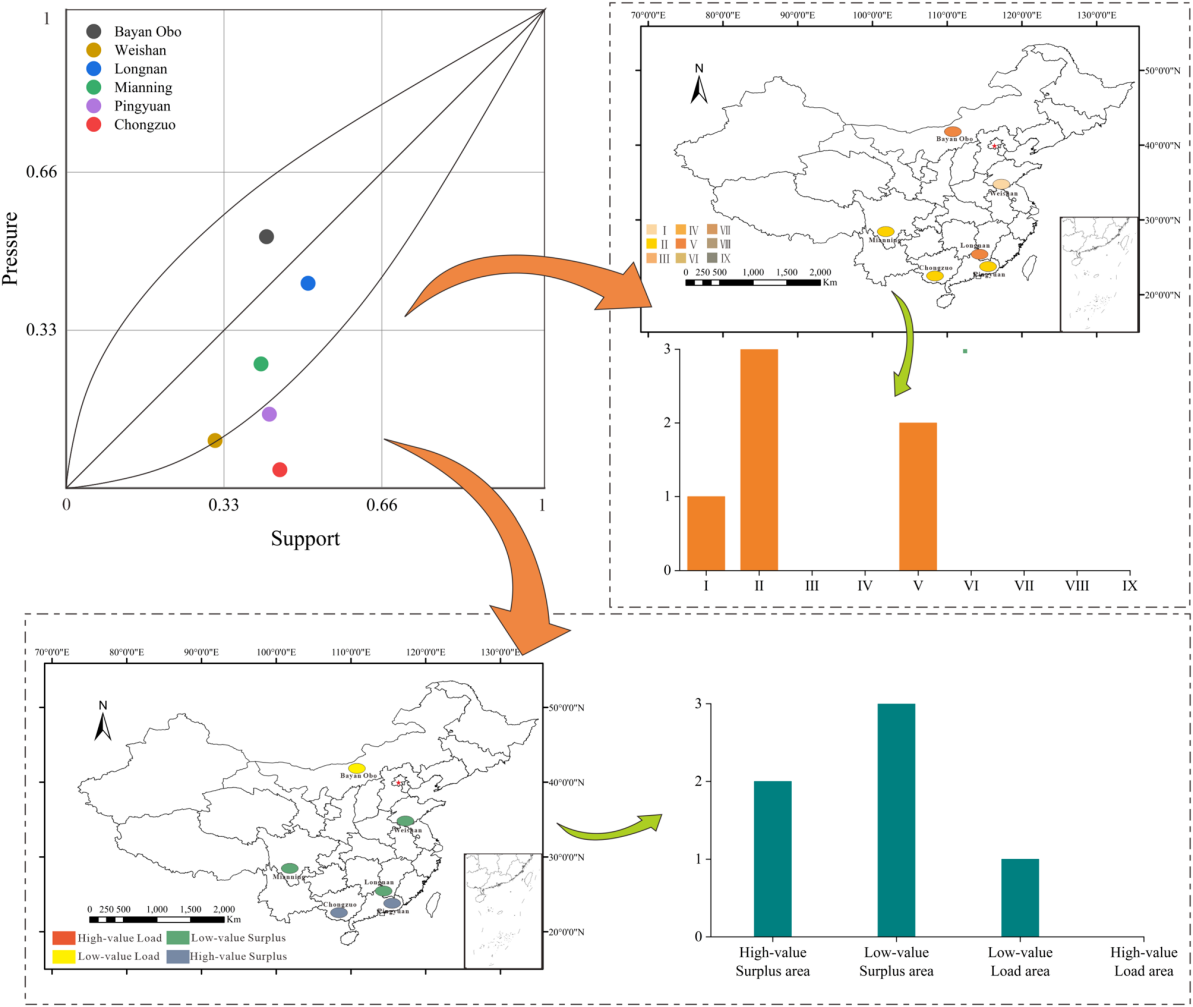
Schematic diagram of the classification of RECC in mining areas.

The model was used to calculate the support index, pressure index and RECC index of the rare earth element mining areas to graphically show the variation in RECC within a mining area (Fig. 4).

In 2019, the Bayan Obo mine area was a low-value load area. The Weishan mine area was a low-value surplus area. The Mianning mine was a low-value surplus area. The Longnan mine was a low-value surplus area. The Pingyuan mine was a high-surplus area. The Chongzuo mine was a high-value surplus area.

The high support index for Longnan and Bayan Obo stems from the amount of rare earth element reserves, the number of rare earth element-related employees and the rare earth element policy. The ionic rare earth element reserves in the Longnan mine account for 36% of China's proven reserves, while Bayan Obo's industrial reserves of rare earth element reserves account for 83.7% of the country's industrial reserves^49^. This large reserve base has strongly supported the high-quality development of the industry and facilitated the development of employment opportunities and subsidiary industries from a more complete industrial chain competitive advantage. The favorable climatic conditions are the reason for the high support index of the Chongzuo mining area. The low amount of water resources per capita was the major reason for the low Weishan support index. This scenario is primarily due to the water resources of Jining city, where the Weishan mine is located. Due to the large temporal and spatial differences in precipitation and poor natural connectivity of water systems, Jining is characterized by "many rivers, few floods, uneven abundance and overall water shortage". Investing to improve the per capita water resources and other ecological indicators in Weishan can rapidly improve the regional support index.

The pressure index of each mining area was ranked from high to low: Bayan Obo > Longnan > Mianning > Pingyuan > Weishan > Chongzuo. The high-pressure index at Bayan Obo was mainly due to the losses from environmental pollution and ecological damage, where the losses from environmental pollution greatly exceeded other mining areas. In recent years, more than 50% of China's rare earth elements have been mined annually at Bayan Obo, inevitably resulting in more serious ecological damage. Ecological damage loss is the main factor contributing to the high-pressure index in the Longnan mining area, with scores that greatly exceed those of other mining areas. In terms of rare earth element types, Bayan Obo, Mianning and Weishan contain light rare earth elements, while Longnan, Pingyuan and Chongzuo contain medium and heavy rare earth elements. The losses from ecological damage when mining light rare earth elements are less than those when mining medium and heavy rare earth elements. Light rare earth ores are mostly mined in northern China, where the terrain is mostly plains, and the vegetation is relatively sparse. Most of the medium and heavy rare earth elements are mined in southern China, and the terrain is dominated by hills and mountains. However, the environmental pollution losses caused by light rare earth element mining were greater than those caused by medium and heavy rare earth element mining. Light rare earth element mines were dominated by atmospheric pollution and radioactive pollution, while medium and heavy rare earth element mines were dominated by water pollution. This result was caused by the different rare earth element smelting methods.

### Analysis of changes in RECC trends at the mining sites

**Figure 5:**
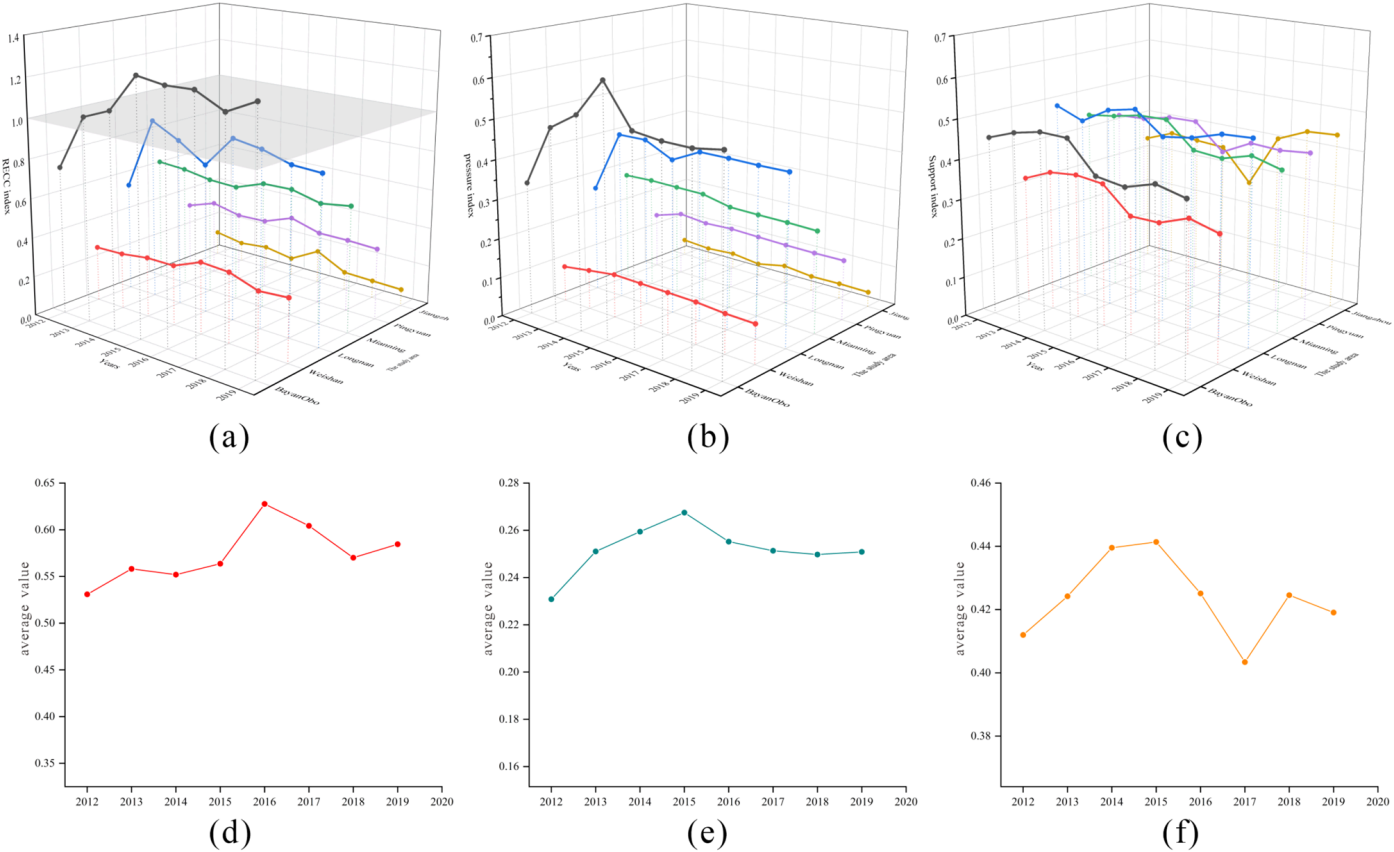
Changes in RECCs for each mine. (**a**) The trend of the RECC index in the mining area; (**b**) The trend of the pressuring index in the mining area; (**c**) The trend of the supporting index in the mining area; (**d**) The trend of average RECC index; (**e**) The trend of average pressuring index; (**f**) The trend of average supporting index.

To more accurately determine the changes in the RECC of rare earth element mining areas, a dynamic analysis of the RECC of rare earth element mining areas from 2012 to 2019 was conducted, and the results are shown in Fig. 5.

Bayan Obo's RECC index increased from 0.7498 (2012) to 1.2543 (2019), and Bayan Obo was in category V. The support index decreased from 0.4510 (2012) to 0.4188 (2019), while the pressure index increased from 0.3382 (2012) to 0.5253 (2019). In 2019, among all the mining areas, the Bayan Obo mining area had the largest stress index (0.5253), and it gradually moved toward the VIII category. Weishan's RECC index rose from 0.2808 (2012) to 0.3647 (2016) before falling to 0.3191 (2019). The Weishan mine was a category II from 2013–2015 and returned to a category I from 2016–2019, with slight fluctuations in the support index and a stable and concentrated trend in the pressure index. The RECC index for the Mianning mine rose from 0.6338 (2012) to 0.6510 (2017) and fell back to 0.6373 in 2019. Mianning was a category II, and the support index increased from 0.4451 (2012) to 0.4731 (2015) and then decreased to 0.4073 (2019). The stress index decreased from 0.2821 (2012) to 0.2596 (2019).

Longnan's RECC index increased from 0.5575 (2012) to 0.8467 (2019), with a peak in 2016 (0.9212), and Longnan was a category II in only 2012 and a category V in the remaining years. The support index increased from 0.4894 (2012) to 0.5056 (2019), while the pressure index increased from 0.2729 (2012) to 0.4281 (2019). Both the support and stress indices were increasing and predicted to be in category V in the longer term. Pingyuan's RECC index increased from 0.3393 (2012) to 0.3637 (2019), with a peak in 2016 (0.4124), and Pingyuan's RECC index increased from 0.4241 (2012) to 0.4471 (2015) and then decreased to 0.4247 (2019). The pressure index increased from 0.1439 (2012) to 0.1606 (2015) and then decreased to 0.1545 (2019). Chongzuo's RECC index decreased from 0.1253 (2012) to 0.0860 (2019), with a peak in 2016 (0.1709). Chongzuo was a category I only in 2012 and a category II in the remaining years. The support index decreased from 0.3381 (2012) to 0.0.2763 (2015) and then rose to 0.4468 (2019). The pressure index decreased from 0.0424 (2012) to 0.0324 (2015) and then increased to 0.0384 (2019). Both the support and pressure indices showed a decreased and then increased state.

### Screening key indicators to improve the support index using the obstacle degree model

**Figure 6:**
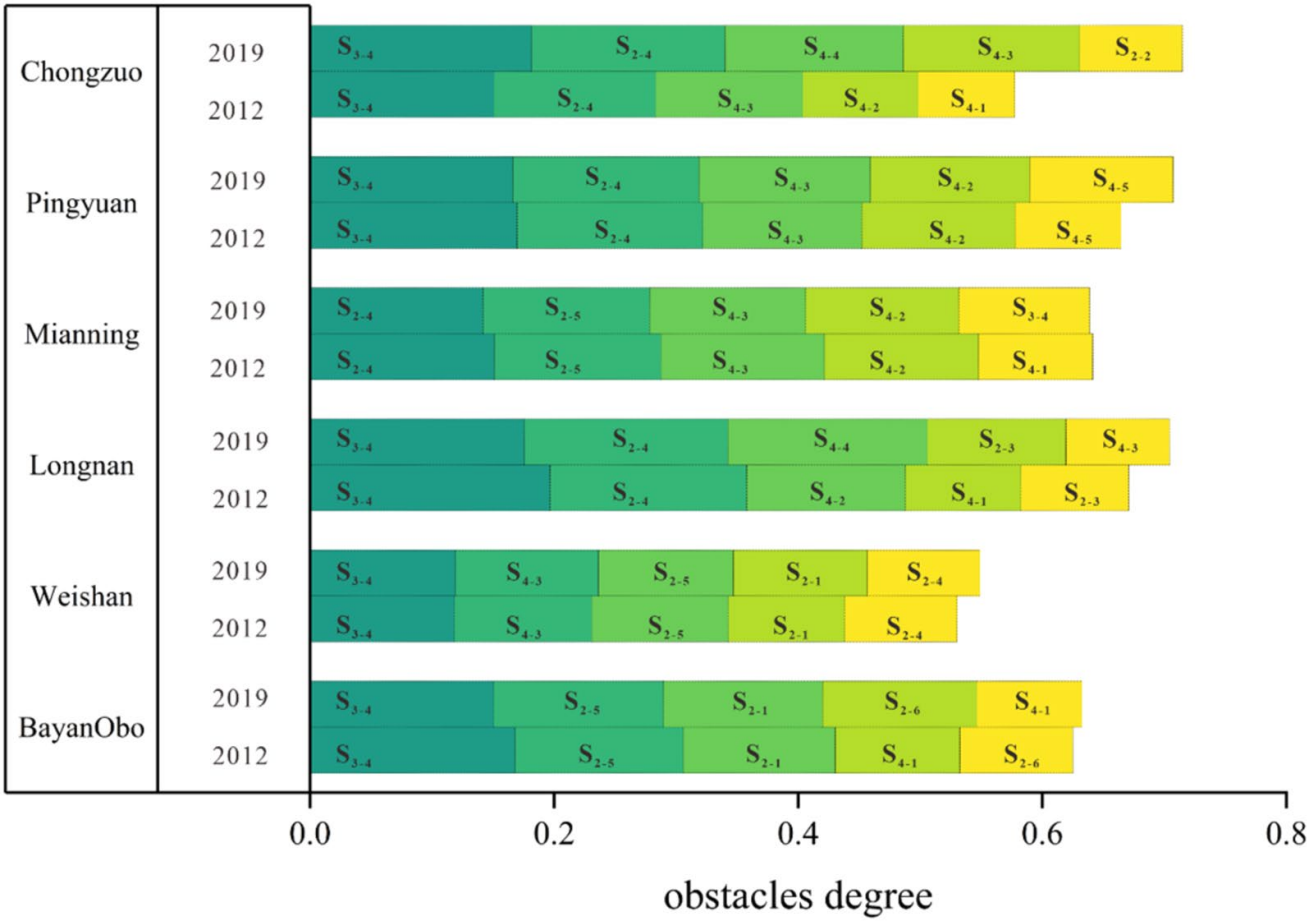
Top 5 obstacle indicators for the support index of the mines.

The analysis of the support surface of each mine area was carried out based on the obstacle degree calculation method. The obstacle degree of each indicator of the mine area was obtained. The five indicators with the largest obstacle degree were selected and arranged from left to right, as shown in Fig. 6. The total obstacle degree of the five indicators of each mine area was > 50%.

The ratio of investment in environmental pollution control to GDP, GDP per capita, and rare earth element reserves were common factors limiting the sustainable development of the mining areas. In 2012, the ratio of investment in environmental pollution control to GDP (5 times), rare earth element reserves (5 times), GDP per capita (4 times), the proportions of medium and heavy rare earth elements (4 times), total tourism revenue (4 times), number of rare earth element-related employees (4 times), total annual precipitation (2 times), water resources per capita (1 time), and forest grassland coverage (1 time) were the main obstacles to improving the support capacity of the mining areas. In 2019, the environmental pollution control investment to GDP ratio (6 times), rare earth element resource reserves (5 times), number of rare earth element-related employees (5 times), GDP per capita (4 times), medium and heavy rare earth element shares (4 times), total annual precipitation (2 times), water resources per capita (1 time), and forest grassland coverage (1 time) were the main obstacles to improving the support capacity of the mining areas. The indicators that appeared more frequently belong to the economic development system and the resource system, which indicates that the common barriers constraining support of most cities were the existence of the economic development system and the resource system. The low ratio of investment in environmental pollution management to GDP was a common problem limiting the achievement of sustainable development in the mining areas. Although the importance of environmental protection in China has been increasing and investment in environmental management has been growing steadily, the ratio of investment in environmental pollution to GDP is still at a relatively low level.

The obstacle degrees of the primary indicators were obtained by summing the obstacle degrees of the secondary indicators. The obstacle degrees for the four support subsystems were calculated (Table 5).

**Table 5:** Obstacle degree and standard deviation of each mine subsystem.

Criteria layer		Climate	Resources	Environment	Economic development
Mining area	Bayan Obo	0.2137	0.3998	0.1860	0.2004
	Weishan	0.1073	0.4469	0.1403	0.3055
	Longnan	0.0582	0.3786	0.2225	0.3406
	Mianning	0.0489	0.4425	0.1029	0.4057
	Pingyuan	0.0241	0.4051	0.2036	0.3672
	Chongzuo	0.0069	0.3888	0.2546	0.3498
Standard deviation		0.0701	0.0341	0.0563	0.0755

The improvement in the support index of Bayan Obo, Weishan, Longnan and Pingyuan was affected. Economic development was the major obstacle to improving the Mianning Support Index. Furthermore, the standard deviation of the climatic conditions was 0.0701, with the climatic conditions varying widely from mine to mine depending on their geographical location. The standard deviation of socioeconomic pressures was 0.0755, which far exceeded the standard deviations of the other factors, and thus, it can be concluded that there was heterogeneity in the economic development of the different mining areas, which affected the RECC of the mining areas.

## Discussion

China's rare earth elements are widely distributed and divided based on rare earth element formulations. The Bayan Obo, Mianning and Weishan mines are abundant with light rare earth elements, and the Longnan, Pingyuan and Chongzuo mines are abundant with medium and heavy rare earth elements, with different element endowment phases in each mining area^50^. Light rare earth elements are mainly in the northern region, and most can be mined at a large scale; however, the mining and smelting processes have a large impact on the environment, and the extraction costs are high^51^. The order of ecological damage loss was Longnan > Pingyuan > Mianning, and Weishan had the least amount of loss. In terms of obtaining rare earth element types, heap leaching and pool leaching processes were mainly used for medium and heavy rare earth element development in the past^52^. Both processes include topsoil stripping and element mining, thus causing more damage to the ecological environment; after switching to in situ leaching, the vegetation of the ore body is not destroyed, and no topsoil is stripped, thus causing less damage to the ecological environment^53^. The government should consider a reasonable layout of mines, especially in the medium and heavy rare earth element areas in the south, and all mineral resources in the key exploration planning areas should undergo ecological and environmental assessments and economic benefit assessments before development to determine the suitability of mining operations based on the results. It is worth noting that the Weishan mine is currently mined underground, and the mine has been using the shallow hole retention mining method for many years, with tailings filling the void area afterward; thus, the ecological damage loss here is small.

The amount of environmental pollution loss from rare earth element smelting was related to the characteristics of their resources, production process and production scale; the following is the order of environmental treatment costs for the mines: Bayan Obo > Mianning > Longnan > Weishan > Chongzuo > Pingyuan. Northern rare earth element mines are mainly dominated by atmospheric and radioactive contamination^54^, and medium and heavy rare earth element mines are dominated by pollution of water and agricultural soils^55,56^. Northern light rare earth elements are mostly polymetallic-associated ores with complex compositions and large tailings, causing serious pollution, of which radioactive pollution is particularly important. Due to its natural decay characteristics, thorium is highly radioactively toxic; insoluble thorium can enter the human body in the form of dust, and these radioactive compounds gradually accumulate in the lungs, and directly damage the lungs^53^. Due to the poor resource conditions, scattered distribution, low abundance, and difficulties scaling up production that the medium and heavy rare earth element mines experience, their use of the ammonium sulfate in situ leaching method will produce a large amount of high concentration ammonia nitrogen wastewater, causing serious pollution to local water resources^57^.

National policies related to rare earth elements are important factors that influence the index of each mining area. Since the beginning of the twenty-first century, China has made up an absolute share of the supply side of the rare earth resource market. However, due to the large number of domestic production enterprises, the competition among enterprises is intense. There is also very serious illegal mining of rare earth elements and theft, and informal mining methods not only seriously pollute the environment but also disrupt the market order. Oversupply is the largest problem that the international rare earth element market is currently facing. This resulted in China supplying most of the rare earth elements to the world, consuming a large number of resources, and bearing substantial environmental costs without reaping the corresponding economic benefits. In response to these problems, the state should take measures to guide the domestic market. First, we should raise the market access threshold to curb low-end excess capacity^58^. The management of rare earth element reserves can be refined, from the general management of light and heavy mining areas to management based on rare earth element allocation, based on the elements. Second, we should increase the application of rare earth element research, expand consumer demand, and fundamentally address the problem of supply exceeding demand in the market. Finally, it is necessary to determine the minimum required indicators for developing and utilizing rare earth elements, to implement differentiated environmental management policies and establish an ecological civilization evaluation mechanism, to introduce a GEP accounting system and to incorporate ecological benefits into the evaluation system.

## Conclusion

This paper studies the RECC status of major rare earth element mining areas in China in 2019 by coupling the support index and pressure index. In addition, this paper studies the dynamics of the RECC for each mining area from 2012 to 2019. Finally, a comparative analysis of the main obstacles supporting the improvement of the index for each mining area in 2012 and 2019 is presented. The main conclusions are as follows: first, the combined weight of ecological damage losses was the largest in the pressure system and the smallest in the social pressure system. The combined weight of climatic conditions in the support system was the largest, and the economic development was the smallest. Second, Bayan Obo was overloaded, and the pressure of human and social activities exceeded the capacity of local resources and environmental services to support them. The RECCs at Longnan and Mianning were very close to the alert level. All other mining areas had surplus RECCs. Third, from 2012–2019, the average RECC index for the six mining districts trended upward, with an inverted V-shaped change in the average pressure and support indices. The increased rate of the pressure index slowed, but the decrease in the support index was larger. Fourth, the ratio of environmental pollution control investment to GDP is the most common problem that most mining communities face in improving their support index. Among all rare earth element mining areas, the light rare earth element mining areas have the greatest environmental pollution losses, and the ecological damage losses in the medium and heavy rare earth element mining areas were greatest.

## Supplementary Information

Supplementary Information.
