# Supplementary material for: Evaluation of resource and environmental carrying capacity in rare earth mining areas in China
Source: Sci Rep. 2022 Apr 12;12:6105. doi: 10.1038/s41598-022-10105-2 (PMC9005666; doi:10.1038/s41598-022-10105-2)
Supplement: Supplementary file 1 — Supplementary Information. [file 41598_2022_10105_MOESM1_ESM.docx]

**Evaluation of the resource and environmental carrying capacity in rare earth element mining areas in China**

Jingjing Bai^a^, Xin Xu^a^, Yaoting Duan^a^, Guangyu Zhang^a^, Zhe Wang^a,b^, Lu Wang^c,*^, Chunli Zheng^a,b,*^

a School of Energy and Environment, Inner Mongolia University of Science and Technology, Baotou 014010, Inner Mongolia, China

b Engineering Research Center of Evaluation and Restoration in the Mining Ecological Environments, Inner Mongolia University of Science & Technology, Baotou 014010, Inner Mongolia, China

c Ganjiang Innovation Academy, Chinese Academy of Sciences, Ganzhou 341000, Jiangxi, China

*Correspondence:

Lu Wang: lwang@gia.cas.cn

Chunli Zheng: zhengchunli1979@163.com

# Contents

[Contents 2](#_Toc93843626)

[1. Literature reviews of RECC in mining areas 3](#_Toc93843627)

[2. Introduction of ecological carrying capacity 11](#_Toc93843628)

[3. Studies on rare earth elements 14](#_Toc93843629)

[4. Research method 15](#_Toc93843630)

[5. Accounting for ecological damage losses in rare earth mining. 17](#_Toc93843631)

[6. Environmental pollution control cost accounting. 18](#_Toc93843632)

[7. Weights of the thirty evaluation factors. 20](#_Toc93843633)

[8. Primary data of RECC of typical rare earth mining areas in China 22](#_Toc93843634)

[9. Analysis data of the obstacles of typical rare earth mining areas in China 29](#_Toc93843635)

[10. Mine RECC Index in 2012-2019. 34](#_Toc93843636)

[References 35](#_Toc93843637)

# 1. Literature reviews of RECC in mining areas

An extensive literature review of existing ecological carrying capacity indicator systems was conducted through an Internet search. These literatures were considered to be scientifically sound and representative in the discipline. The analysis was also done from the perspective of publication year, number of citations, research direction, document type, country/region of literature origin and article overview. The selection of recommended indexes from these representative studies was justified as candidate indexes. The availability of indexed data was also considered by referencing these indexes to statistical data sources.


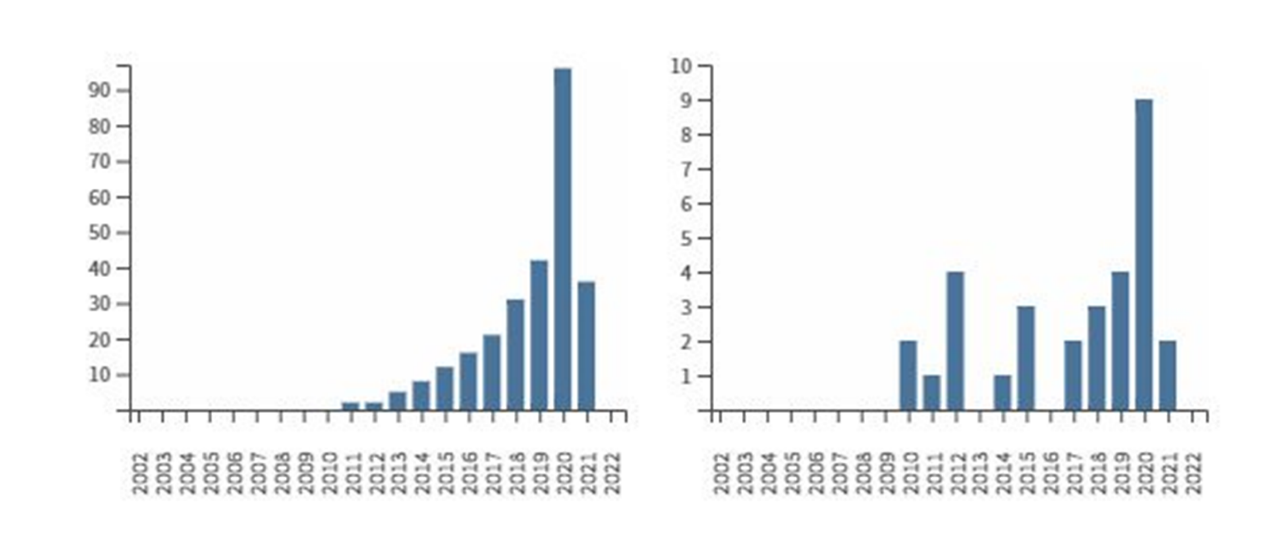


Fig. S1. Total Publications by Year Fig. S2. Sum of Times Cited by Year


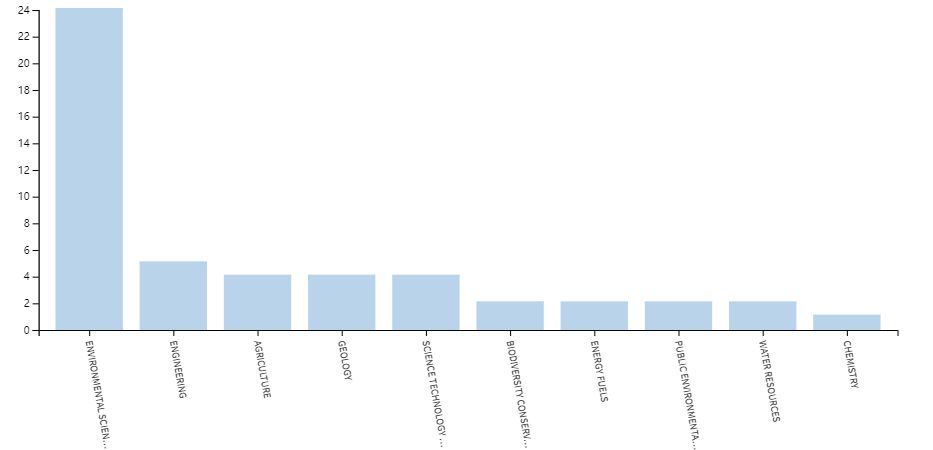


Fig. S3. Analysis of research direction


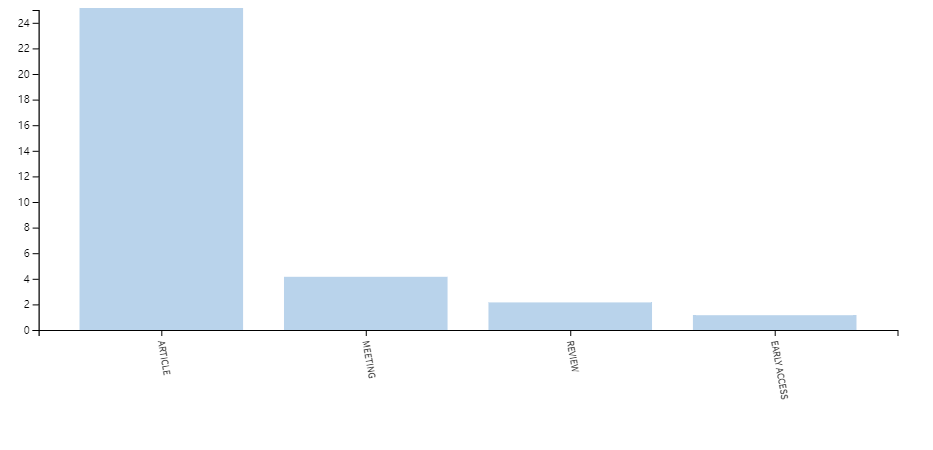


Fig. S4. Document type


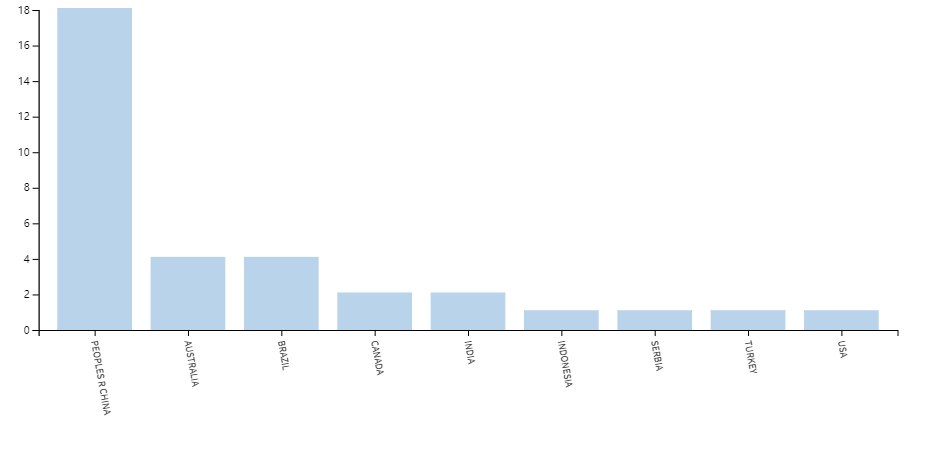


Fig. S5. Country/region of literature source

Table. S1. A review of ecological carrying capacity in mining areas

| **References** | **Type** | **Title** | **Brief description of content** |
| --- | --- | --- | --- |
| 1 | Article | The effects of land subsidence and rehabilitation on soil hydraulic properties in a mining area in the Loess Plateau of China | To analyze the effects of land subsidence and rehabilitation on soil hydraulic properties, an underground coalmine in the Loess Plateau of China was selected to conduct a field plot experiment. |
| 2 | Article | Drought severity-duration-frequency curves: a foundation for risk assessment and planning tool for ecosystem establishment in post-mining landscapes | In the context of mined land rehabilitation quantifying the severity-duration-frequency (SDF) of droughts is crucial for successful ecosystem rehabilitation to overcome challenges of early vegetation establishment and long-term ecosystem resilience. |
| 3 | Review | Ecological Potential of Plants for Phytoremediation and Ecorestoration of Fly Ash Deposits and Mine Wastes | This review presents a current knowledge in phytomanagement of fly ash deposits, mine waste-rock and tailings. |
| 4 | Article | Assessing the development of rehabilitated grasslands on post-mined landforms in north west Queensland, Australia | Annual vegetation monitoring was carried out at two restoration sites and selected reference areas in the coal mine area. |
| 5 | Article | Restoration of rare earth mining areas: organic amendments and phytoremediation | In this study, experiments involving dry grass landfilling, chicken manure broadcasting, and plant cultivation were carried out to reclaim a rare earth mine area located in Heping County, Guangdong Province, China. |
| 6 | Article | Mining Communities from a Resilience Perspective: Managing Disturbance and Vulnerability in Itabira, Brazil | this study describes the various components of the Itabira social-ecological system revealing the challenges brought about by mining's dominance. |
| 7 | Article | Response of ecological storage and conservation to land use transformation: A case study of a mining town in China | Taking Cishan Town, a mining town in China, as a case study, this paper estimates the "past-present-future" ecological storage impacted by the process, result and possibility of land use transformation. |
| 8 | Article | Long-term data suggest jarrah-forest establishment at restored mine sites is resistant to climate variability | We built a structural equation model to discriminate the relative effects of climate, restoration practice, and their interactive effects on three response variables including species richness of the restored plant assemblages. |
| 9 | Article | Ecological resilience assessment of an arid coal mining area using index of entropy and linear weighted analysis: A case study of Shendong Coalfield, China | In this paper, Shendong coal field, one of the high-strength coal mining areas in western China is used as a research area. An O-U (Overground-Underground) analysis framework and model was established to quantitatively assess the degree of mining impact on the eco-system and its resilience. |
| 10 | Article | Assessment and analysis of noise levels in and around lb river coalfield, Orissa, India | A study was carried out to assess the noise level in different opencast projects in and around Belpahar and Brajarajnagar areas of lb river coalfield. |
| 11 | Article | Assessment of Landscape Ecological Health: A Case Study of a Mining City in a Semi-Arid Steppe | The purpose of this study was to construct a LEH assessment index system and evaluate the LEH of a mining city located in a semi-arid steppe. |
| 12 | Article | A quantitative assessment of vulnerability using social-economic-natural compound ecosystem framework in coal mining cities | In this study, based on the analysis of the social-economic-natural compound ecosystem (SENCE), we constructed a vulnerability evaluation indicator system (28 indicators) for coal mining cities. |
| 13 | Article | Zoning and management of phreatic water resource conservation impacted by underground coal mining: A case study in arid and semiarid areas | phreatic water resource management methods are proposed based on water resource utilization, coal mining methods, and water-resistant layer reconstruction. |
| 14 | Article | Resource recovery as a sustainable perspective for the remediation of mining wastes: rehabilitation of the CMC mining waste site in Northern Cyprus | This paper highlights resource recovery and stabilization as the novel approach adopted in the rehabilitation strategy of the abandoned copper mine site (CMC mine) located in Northern Cyprus. |
| 15 | Review | Ecological methods and indicators for recovering and monitoring ecosystems after mining: A global literature review | There was a significant increase of articles along time approaching the use of geotechnologies and arbuscular fungi. |
| 16 | Article | Regime shift and redevelopment of a mining area's socio-ecological system under resilience thinking: a case study in Shanxi Province, China | Adopting the perspective of resilience thinking, this study investigated the historical dynamics of a post-mining area in Shanxi Province and considered its future development. |
| 17 | Article | Prairie Grass Establishment on Calcareous Reclaimed Mine Soil | The establishment of 15 prairie grasses as monocultures in mine reclaimed soils in southeastern Ohio was evaluated in May 2005 and May 2006. |
| 18 | Article; Early Access | Restoration of mine degraded land for sustainable environmental development | Selective plantation and application of various soil amendments along with the topsoil potentially enhance the recovery of mine degraded lands. |
| 19 | Article | Application of an evaluation method of resource and environment carrying capacity in the adjustment of industrial structure in Tibet | This study develops a conceptual framework for resource and environment carrying capacity estimation to support the co-development planning of industries, population and resources & environment. |
| 20 | Article | Quantitative Evaluation of the Eco-Environment in a Coalfield Based on Multi-Temporal Remote Sensing Imagery: A Case Study of Yuxian, China | Quantitative Evaluation of the Eco-Environment in a Coalfield Based on Multi-Temporal Remote Sensing Imagery: A Case Study of Yuxian, China |
| 21 | Article | Analyzing Ecological Functions in Coal Mining Cities Based on RS and GIS | This paper chooses Qitaihe City as a typical study area to expatiate the ecological function of mining cities from two aspects of landscape ecology and ecological carrying capacity. |
| 22 | Article | Increasing green infrastructure-based ecological resilience in urban systems: A perspective from locating ecological and disturbance sources in a resource-based city | This paper combines source-sink landscape theory, on the basis of considering ecological processes, and builds a framework of factors that affect the ecological resilience of resource-based cities. |
| 23 | Article | Composition and diversity of prokaryotes at an iron ore post-mining site revealed the natural resilience 10 years after mining exploitation | Prokaryotes play crucial roles in the rehabilitation process to restore the ecological integrity of disturbed areas. |
| 24 | Proceedings Paper | Environment Carrying Capacity Evaluation of Coal Mining in Shanxi Province | This paper established a comprehensive evaluation model for environmental impact of coal mining through three aspects of ecological stability, resource and environment carrying capacity, coal mining pressure with analytic hierarchy process (AHP). |
| 25 | Article | Determining the scale of coal mining in an ecologically fragile mining area under the constraint of water resources carrying capacity | In view of the lack of water resources in arid and semi-arid areas, this paper studies the scale of coal mining in arid and semi-arid areas under the constraint of the water resources carrying capacity (WRCC) with the aim of realizing the conservation mining of ecological environment. |
| 26 | Article | Resilience Perception of a Mining Town in Eastern Amazonia: A Case Study of Canaa Dos Carajas, Brazil | This paper analyzes, in the context of mining, the perception of the resilience of Canaa dos Carajas population in Para State, Brazilian Amazon. |
| 27 | Article | Estimation of the Restored Forest Spatial Structure in Semi-Arid Mine Dumps Using Worldview-2 Imagery | The purpose of this study was to assess the feasibility of estimating the SSPs of restored forest in semi-arid mine dumps using Worldview-2 imagery. |
| 28 | Article | Ecological vulnerability assessment and spatial pattern optimization of resource-based cities: A case study of Huaibei City, China | We used Huaibei City, one of the representative coal resource-based cities, as a case study for measuring ecological vulnerability. |
| 29 | Proceedings Paper | Sustainability for Management and Protection Tin Mining Environment | During the exploitation is done are not beyond the limits of resources support neighborhood then natural resources can be used sustainably in accordance carrying capacity, i.e. the ability of natural resources to support the life of all beings in the ecosystem on an ongoing basis. |
| 30 | Proceedings Paper | Choice and Application of Governance Mode of Mining Subsidence Areas in Cities | Taking Tangshan City as an example, this paper analyzes the feasibility of the global ecological management model through the analysis of the ecosystem carrying capacity before management, and evaluates its management effect from different angles. |
| 31 | Proceedings Paper | Measurement on Environmental Carrying Capacity of Coal Industry: Shaanxi as an Example | On the basis of the predecessors study about environmental carrying capacity, this paper builds an environmental pressure-supportive relationship model, quantizes the Shaanxi coal industrial environmental stress and supportive capacities by the method of principal component analysis, and measures out the environmental carrying capacity. |

Table. S2. Evaluation index system of the RECC.

| **System** | **Criteria layer** | **Indicators (units)** | | **System** | **Criteria layer** | **Indicators (units)** | |
| --- | --- | --- | --- | --- | --- | --- | --- |
| Support | Climate Conditions | S_1-1_ | Frost free period (days) | Pressure | Ecological damage loss | P_1-1_ | Loss of ecological value volume of organic matter due to rare earth mining Ten thousand yuan） |
|  |  | S_1-2_ | Annual average relative humidity (%) |  |  | P_1-2_ | Rare earth mining leads to the loss of value quantity of released O^2^ and fixed CO^2^ (Ten thousand yuan) |
|  |  | S_1-3_ | Annual average temperature (℃) |  |  | P_1-3_ | Rare earth mining leads to the loss of water conservation value amount (Ten thousand yuan) |
|  | Resource Endowment | S_2-1_ | Total annual precipitation (mm) |  |  | P_1-4_ | Rare earth mining leads to the loss of soil conservation value amount (Ten thousand yuan) |
|  |  | S_2-2_ | Arable land to regional area (%) |  |  |  |  |
|  |  | S_2-3_ | Forest-grassland coverage (%) |  | Environmental pollution loss | P_2-1_ | Rare earth smelting water pollution treatment cost accounting (Ten thousand yuan) |
|  |  | S_2-4_ | Rare earth resources reserves (million tons) |  |  | P_2-2_ | Rare earth smelting air pollution treatment cost accounting (Ten thousand yuan) |
|  |  | S_2-5_ | Rare earth resources reserves (million tons) |  |  | P_2-3_ | Rare earth smelting solid waste pollution treatment cost accounting (Ten thousand yuan) |
|  |  | S_2-6_ | Water resources per capita (m^3^) |  |  | P_2-4_ | The radioactivity (nGy/h) |
|  | Environmental Governance | S_3-1_ | Comprehensive utilization rate of industrial solid waste (%) |  |  |  |  |
|  |  | S_3-2_ | Urban sewage treatment rate (%) |  | Social pressure | P_3-1_ | Urban registered unemployment rate (%) |
|  |  | S_3-3_ | Harmless treatment rate of domestic waste (%) |  |  | P_3-2_ | Share of secondary industry in GDP (%) |
|  |  | S_3-4_ | Environmental pollution control investment to GDP ratio (%) |  |  | P_3-3_ | Urban per capita daily domestic water consumption (L) |
|  | Economic Development | S_4-1_ | Foreign exchange earnings from tourism (USD million) |  |  | P_3-4_ | Natural population growth rate (%) |
|  |  | S_4-2_ | GDP per capita (RMB) |  |  | P_3-5_ | Energy consumption of 10,000 Yuan GDP (t standard coal) |
|  |  | S_4-3_ | Number of Rare Earth Related Employees (Number) |  |  | P_3-6_ | Annual mining volume (million tons) |

The indicators affecting ecological carrying capacity can be divided into two main types: basic indicators and characteristic indicators of ecological carrying capacity. Among them, the basic indicators are mainly some common factors that affect the ecological carrying capacity. In this study, by collecting the indicators used by previous people to evaluate the ecological carrying capacity, we find the common indicators among them and take them as the basic indicators of the ecological carrying capacity of the research area; the characteristic indicators are mainly selected to represent some special indicators for the different research areas, such as this paper studies the rare earth mining area, so the comprehensive utilization rate of industrial solid waste, radioactivity and so on are selected. In addition, because there are more indicators affecting ecological carrying capacity and the indicator system is huge, it will take a lot of time and energy to collect data and calculate ecological carrying capacity, and the influence of such indicators on the comprehensive score of ecological carrying capacity is not obvious in the final calculation, so in the actual study, the principle of "cut-off" is adopted, that is, the influence of the indicator on Therefore, the "cut-off" principle is adopted in the actual study, that is, such indicators with less than 1% impact on ecological carrying capacity can be disregarded. However, it should be noted that, because different regions have their special characteristics, when using this principle to select indicators, it is necessary to combine the characteristics of the study area, so as to avoid deleting some important indicators by mistake, which makes the results unreliable. For example, the indicator of "radioactivity" has little impact on the ecological carrying capacity of general areas, but it is very important for rare earth mining areas, so it cannot be deleted. Finally, it should be noted that for indicators that are difficult to collect, similar indicators with higher relevance can be selected instead, which saves manpower and material resources and does not have a large impact on the final results. For example, some relative indicators (per capita water use rate, which is related to the total amount of water resources, population size, time of use, etc.) are not easy to collect, and can be replaced by their corresponding absolute indicators (per capita water use), or replaced by similar indicators that are correspondingly easy to collect. At the same time, it is necessary to avoid indicators with high correlation in the indicator system (e.g. average annual and average monthly precipitation), which not only increase the workload, but also interfere with the later analysis.

It should be mentioned that since Bayan Obo is located in Inner Mongolia Autonomous Region and the area of grassland is large in Inner Mongolia Autonomous Region, it has been the basis for Mongolian nomadic people to flourish, while the forest coverage in other study areas is large and the area of grassland is insignificant, therefore, the "area of grassland in the region" and "forest-grassland coverage" are combined as one of the basic indicators. Forest coverage" is combined into "forest and grassland coverage" as one of the basic indicators; as rare earth smelting produces a lot of pollution, the pollution in rare earth mining areas is more serious, and it is obviously incomplete to consider only the three waste emissions without considering the level of pollution control, so "the ratio of investment in environmental pollution control to GDP" will be included. environmental pollution control investment to GDP ratio" as one of the characteristic indicators; since monazite is one of the carrier minerals of rare earth elements, and monazite is rich in thorium, this radioactive element will have a great impact on ecology, environment and society, so "radioactivity" is one of the characteristic indicators.

As a subsystem of nature, the ecosystem contains many elements that provide available resources and ecological services for human production and life, and absorbs pollution and waste generated by human activities; although social development exerts various pressures on the ecosystem, it can also strengthen the ecosystem carrier through, increasing green areas, protecting water sources and other environmental treatments, and reducing the ecological load through sewage treatment, solid waste comprehensive utilization and increased environmental pollution control to reduce the ecological load. In addition, the improvement of environmental management and technological progress also contributes to the reduction of ecological and environmental loads.

# 2. Introduction of ecological carrying capacity

Carrying capacity was originally described as the maximum individuals that a habit can support in a region^32^.Carrying capacity is determined based on the relationship between a “carrier” or support object, and a “bearing object” The carrying capacity is defined as the carrier's ability to support the “bearing object”^33^.Hadwen and Palmer^34^ considered carrying capacity as the number of lives that can be supported without damaging the ecological environment and emphasized that the ecological environment should not be damaged for the mission of sustainable development. In 1970s, Holling^35^ proposed the concept of ECC, which is the ability of ecosystem to resist external interference and maintain the relative stability of original ecological structure. In line with economic and social development, researchers gradually focus on the impacts of human activities on the ecological environment. Arrow et al.^36^ discussed the relationship between human activities and ECC. Chapman and Byron^37^ noted that overloading of ECC has become a common issue across the world. There still other definitions on the term ECC. For example, Sun et al.^38^ defined ECC as the capability to support the coordinated and sustainable urbanization. Ma et al.^39^ considered ECC as a holistic framework evaluating environment, living beings and their interactions. Whilst various definitions on ECC are presented, in essence, ECC refers to the capacity of sustainable development sup-ported by the various ecological resources.

A comprehensive research theory and method is widely used by many scholars to promote the development of ecological carrying capacity model. Carrying capacity research is mostly carried out by regional nature, and due to the differences of various regions, most of them first analyze the type of carrying capacity, determine the indicators affecting the carrying capacity of resources, establish the evaluation index system, determine the index weights, and then complete the evaluation through various evaluation methods ^40-42^；In the evaluation process, the researcher determines the results of the study in two ways: the first process assumes that resource, environmental, economic, social and other criteria are additive through the positive and negative characteristics of the indicators ^43, 44^；Another process is to construct a pressure-state-response as a system layer ^40, 45^。

Models are usually involved in the calculation of carrying capacity, for example, the severe shrinkage of the Aral Sea and the coordination of water resources in upstream and downstream areas have been evaluated by combining remote sensing (RS) data and geographic information system (GIS) technology ^46^；The dynamic evolution of ecological footprint and ecological carrying capacity in Jiangsu Province was studied based on ecological footprint theory ^47^，The impact of human activities on natural systems is assessed to provide guidance for more efficient and rational allocation of resources. System Dynamics Model ^48^ It is possible to quantitatively analyze the intrinsic relationships between the structure and function of various complex systems, and to quantitatively analyze various characteristics, making it suitable for objective, long-term studies of dynamic trends. TOPSIS model has the advantages of horizontal and vertical comparative analysis and simple calculation, ^49^ evaluated the weights of each index and county RECC in Chongqing City using TOPSIS model. The results showed that the environmental index had the largest weight, followed by the resource index and the socioeconomic index. ^40^ constructs a support index and pressure index to calculate the level of support resources and the level of environmental pressure that human activities induce in 36 municipalities, provincial capitals and sub-provincial cities in China from 2010 to 2016; in addition, this paper analyzes the factors affecting RECC；In recent years, research on carrying capacity has led humans to recognize that environmental disturbances caused by human activities illustrate the fragility and variability of environmental systems in terms of their stability, integrity and ability to withstand external disturbances ^50^，It can easily lead to qualitative or mutational changes in the structure and function of environmental systems. These findings have facilitated the application of mutation sequence models in the study of carrying capacity ^51^。Table S1 summarizes the main research methods and advantages and disadvantages of regional ecological carrying capacity

Table. S3. Summary of research on regional RECC.

| **Research method** | **Reference** | **Research process** | **Research limitations** |
| --- | --- | --- | --- |
| three-dimensional evaluation model | ^43, 52^ | Based on the study of the relationship between environmental quality, ecological quality and social economy, a three-dimensional evaluation model of the carrying capacity of regional ecological environment for social and economic development is established. The three-dimensional cube model is divided into eight panels, representing different levels of socio-economic development and the comprehensive condition of ecological environment quality. | A single quantitative value can only reflect the changes in the strength of the carrying capacity of the resources and environment, and cannot determine whether the pressure of human social activities on the resources and environment has exceeded the carrying capacity of the resources and environment. |
| System dynamics method | ^48, 53, 54^ | Determine the target system and each subsystem; model the system, determine the functional relationships between variables, and simulate the system model. | The selection of parameter indicators is difficult. The relative determinism of the simulation model lags behind the dynamic characteristics of the system, and the calculation process is huge. |
| Entropy method | ^9, 55, 56^ | Objectively determine the weights based on the raw data of the indicators; establish the indicator system. | The importance of the indicators themselves is ignored, and sometimes the determined indicator weights can be far from the expected results, while the entropy value method cannot reduce the number of dimensions of evaluation indicators. |
| Ecological footprint method | ^57-59^ | We quantify land area, calculate supply and demand, and compare supply and demand by supply and demand to forecast its development trend. | There are no specific and feasible criteria for the selection and quantification of indicators to divide the RECC of different regions. |
| Comprehensive evaluation method | ^60-62^ | A multi-level index system was constructed, and the weights were determined by principal component analysis with linear weighting. | Quantitative studies of RECC only cannot determine whether the assessed RECC is overloaded. |
| TOPSIS method | ^63-65^ | Construct the index system, define certain measures in the target space, and calculate the degree to which the target approaches or deviates from the positive or negative ideal solution; evaluate the evaluation results. | Based on subjective and comprehensive evaluation criteria, the classification criteria lack explanatory and persuasive power. |

# 3. Studies on rare earth elements

Intensive rare earth mining and production activities have had a significant impact on the environment and health in China^66^. Mining activities release dust containing rare earth elements, other toxic metals and chemicals into the air and surrounding water bodies, affecting local soil, wildlife, and vegetation in addition to humans^67-70^. More REE mining will mean more environmental degradation and human health hazards, and waste disposals may be exposed to weathering conditions and have the potential to contaminate air, soil and water if proper monitoring and protection measures are not taken^71^. Research on rare earth elements has focused on ecological risk, bioaccumulation, bioremediation, biotoxicity, environmental pollution and quantification of environmental costs, as shown in Table 1. Currently there is still a gap in the horizontal comparative evaluation of RECC of rare earth mining areas. The establishment of a comprehensive resource and environment evaluation index system applicable to rare earth mining areas is beneficial to the integrated development of rare earth resource-based areas through horizontal comparative evaluation.

Table. S4. The main types of research in the direction of rare earth ecology and environment

| **Research Direction** | **References** |
| --- | --- |
| Ecological risks | ^66, 70-73^ |
| Bioaccumulation | ^74, 75^ |
| Bioremediation | ^76-79^ |
| Biotoxicity | ^80, 81^ |
| Environmental pollution and protection | ^69, 82-84^ |
| Quantification of environmental costs | ^73^ |
| Recovery of rare earth elements | ^85^ |
| Life Cycle Inventory of Rare Earth Products | ^86^ |
| The quality control of rare earth element determinations in environmental matrices | ^87, 88^ |

# 4. Research method

**4.1 Construction of standardized evaluation matrix**

Suppose there are m evaluation indicators and n evaluation objects, then the value of indicator j in object i is taken as A_ij_, then the decision matrix of all objects can be expressed as:

| S=$\left[ \begin{matrix} S_{11} & \cdots& S_{1n} \\ \vdots& \ddots& \vdots\\ S_{m1} & \cdots& S_{\mathrm{mn}} \end{matrix} \right]$ | (1) |
| --- | --- |

Since the values of selected indicators in ecological carrying capacity are not uniform in magnitude, in order to solve the problem of uniformity in the magnitude of model parameters, so that the interval of values after eliminating the magnitude with the original data is limited between 0 and 1, it is necessary to standardize the data of different units for further comparative analysis and comprehensive calculation on the same scale. In this paper, the normalization process is carried out using the polar difference standardization method.

| $r_{ij}=\frac{a_{ij}-min(a_{ij})}{max(a_{ij})-min(a_{ij})}$ | (2) |
| --- | --- |

Then, the normalized decision matrix can be obtained as:

| R=$\left[ \begin{matrix} r_{11} & \cdots& r_{1n} \\ \vdots& \ddots& \vdots\\ r_{m1} & \cdots& r_{\mathrm{mn}} \end{matrix} \right]$ | (3) |
| --- | --- |

**4.2 Defining the weighting values for RECC indicators**

A reasonable assignment of weights plays a crucial role in the scientific rationality of evaluation results, and a reasonable assignment method should assign weights to decision indicators based on both the intrinsic laws between indicator data and expert experience. In this study, a comprehensive subjective and objective assignment method is used to solve this problem. Since AHP is the most common and dominant method in the subjective assignment method. In the objective assignment method, the entropy method is the most frequently used, objective and stable assignment method, so the integrated assignment method uses a combination of these two methods.

1. AHP

The analytic hierarchy process (AHP) was formally introduced by American operations research scientists^89^. It was a systematic and hierarchical analysis method combining qualitative and quantitative. The comprehensive evaluation system established in this paper has a hierarchical structure and was well suited to be analyzed by hierarchical analysis.

The judgment matrix was defined as the relative importance of the relevant elements in the hierarchy assuming that the elements in level A are related to the elements in level B. In constructing the matrix, the expert evaluates the importance of factors at the same level based on a 9-point scale (Table 4) for these factors and forms a judgment matrix.

Table S5 Definitions of 9-point scale grades.

| **Value** | **Definition** |
| --- | --- |
| 1 | Factors i and j have the same degree of importance |
| 3 | The importance of factor i is slightly higher than that of factor j |
| 5 | The importance of factor i is significantly higher than that of factor j |
| 7 | The importance of factor i is much higher than that of factor j |
| 9 | The importance of factor i is extremely higher than that of factor j |
| 2, 4, 6, 8 | Median value between the two adjacent values |

Based on the above judgment matrix, the eigenvectors and eigenroots of the matrix are calculated. The maximum eigenvector $w=\left( w_{1},w_{2},w_{3},\ldots,w_{m} \right)$, of the judgment matrix, where the components are the weights of the elements in the target layer. The judgment matrix needs to satisfy the consistency judgment condition (Eq. (4)).

| $CR=\frac{\frac{\lambda_{max}-n}{n-1}}{RI}<0.1$ | (4) |
| --- | --- |

where $\lambda$ is the maximum characteristic root and RI is the average random consistency index; the RI values are shown below (Table 5).

Table S6 RI values.

| n | 1 | 2 | 3 | 4 | 5 | 6 | 7 | 8 | $\cdots$ |
| --- | --- | --- | --- | --- | --- | --- | --- | --- | --- |
| RI | 0 | 0 | 0.58 | 0.90 | 1.12 | 1.24 | 1.32 | 1.41 | $\cdots$ |

n was the judgment matrix order, when CR < 0.1, the consistency of the judgment matrix was acceptable; otherwise, it was otherwise to reconstruct the pairwise judgment matrix until a satisfactory consistency was achieved.

(2) The entropy method

The objective weighting method determines the weights of indicators based on their intrinsic information, which can eliminate artificial interference and make the results more factual. The entropy method was to determine the weight of the index based on the amount of information, which is one of the objective fixed weighting methods^90^. Then, the information entropy of the jth index can be expressed by Eq. (5).

| $e_{j}=-\frac{1}{\ln m}\sum_{i=1}^{m} P_{ij}\ln P_{ij}$ | (5) |
| --- | --- |

Here, n was the number of evaluation levels and$P_{ij}$satisfied $\sum_{j=1}^{m} P_{ij}=1$.When $P_{j}=0$, $e_{j}=0$.Then, the entropy weight of the jth evaluation factor can be expressed as in Eq. (6).

| ${w^{'}}_{j}=\frac{1-e_{j}}{\sum_{j=1}^{m} (1-e_{j})}$ | (6) |
| --- | --- |

The weights of evaluation indexes obtained by AHP and entropy weight method were $w=w_{1},w_{2},w_{3},\ldots,w_{m}$,$w^{'}={w^{'}}_{1},{w^{'}}_{2},{w^{'}}_{3},\ldots,{w^{'}}_{m}$, and the combination weights obtained by using fuzzy comprehensive evaluation method were:

| $W_{i}=0.5{w_{i}+0.5w^{'}}_{i}$ | (7) |
| --- | --- |

# 5. Accounting for ecological damage losses in rare earth mining.

The technical route of ecological damage accounting for rare earth mining is shown in Fig. S6, which mainly includes the definition of ecological damage scope and the accounting of ecological damage in terms of physical and value losses. Firstly, we collect the basic data, and then use the relevant methods to define the ecological damage range, which includes both direct damage range and indirect damage range. Considering the topography, vegetation type, soil type, distance from the mining area and other factors, the spatial analysis function was used to select an area similar to the mining habitat and not disturbed by mining as the base reference area, and the physical quantities of the direct and indirect damage areas and the base reference area were estimated according to the determined ecological damage assessment index. Since the vegetation in the direct damage area of rare earth mining is destroyed, the physical loss of the direct damage area of rare earth mining is determined by the physical amount of the reference area, i.e., the physical amount per unit area of a vegetation type in the reference area multiplied by the area of the vegetation type destroyed by the mining area. The physical loss of the indirect damage range of rare earth mining is determined by the physical quantity of the reference area and the physical quantity of the indirect damage range, that is, the difference between the physical quantity of the reference area and the physical quantity of the indirect damage range is aggregated and determined, and then the physical quantity of ecological damage is estimated, and finally the physical quantity of ecological damage is multiplied by the unit value to obtain the value of economic loss of ecological damage of rare earth mining.


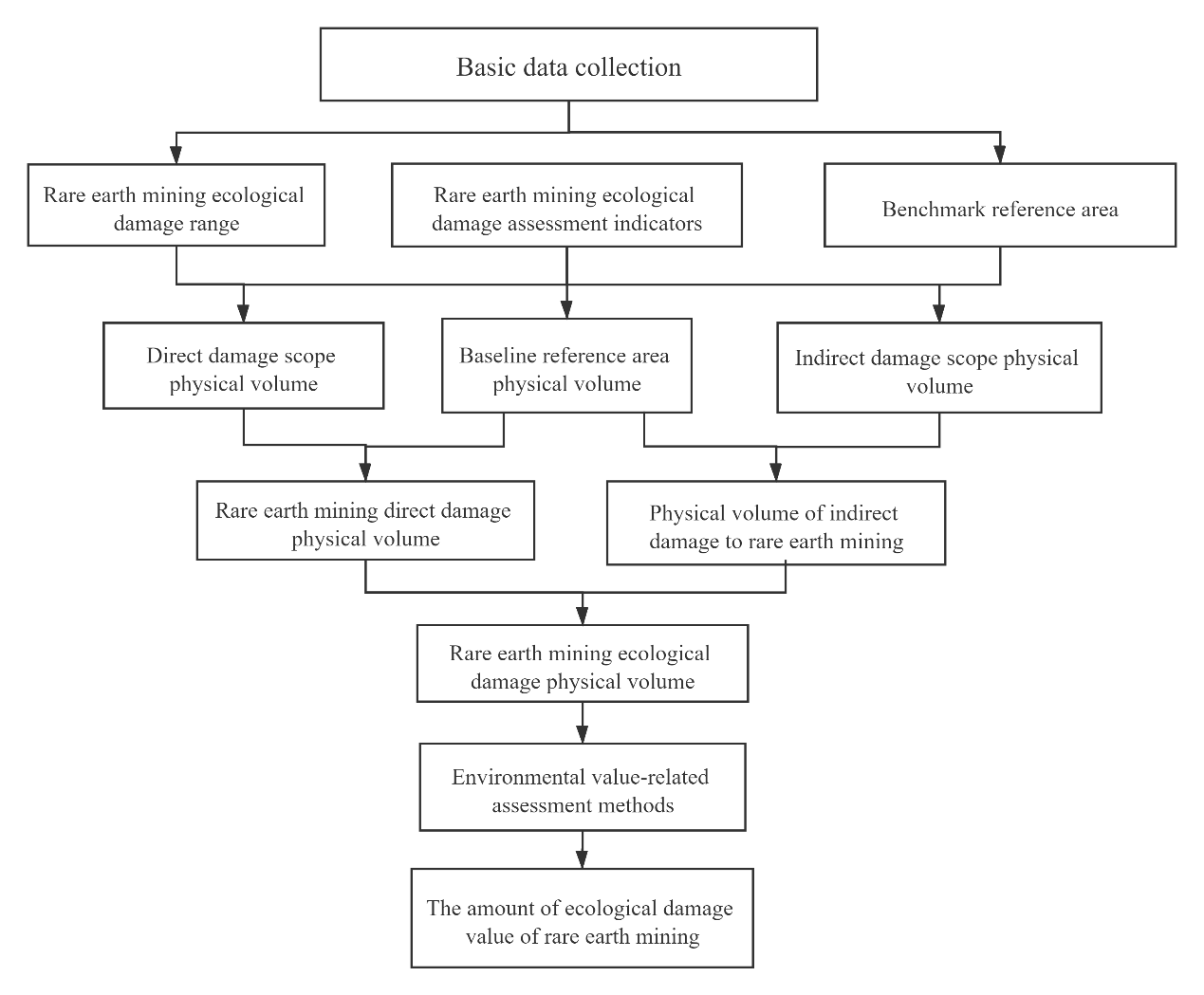
 Fig. S6. The technical route of accounting for ecological damage loss of rare earth mining

# 6. Environmental pollution control cost accounting.

The technical route of environmental pollution treatment cost accounting is shown in Fig. S7, environmental pollutant production and emission accounting using pollution source census data and pollution source census dynamic update data, reference to the "pollution source census production and emission coefficient manual", approved the northern light rare earth and southern medium and heavy rare earth smelting unit REO production and emission coefficients, combined with the amount of REO smelting in different provinces in the last decade, calculate the amount of rare earth smelting in rare earth mining areas resulting in the production and emission of pollutants. emissions.

Environmental pollution treatment cost accounting using pollution source census data, pollution source census dynamic update data, and environmental statistics database, select key typical enterprises, reference to China's "regulations on the collection and use of sewage charges", respectively, calculate the pollution treatment unit pollutant costs of different rare earth products in the treatment and refining process of light rare earths in the north and medium and heavy rare earths in the south, combined with the amount of pollutants generated in the smelting of rare earths in different mining areas, using the pollution The environmental pollution control cost of rare earth smelting in key mining areas is calculated by the cost of control method.


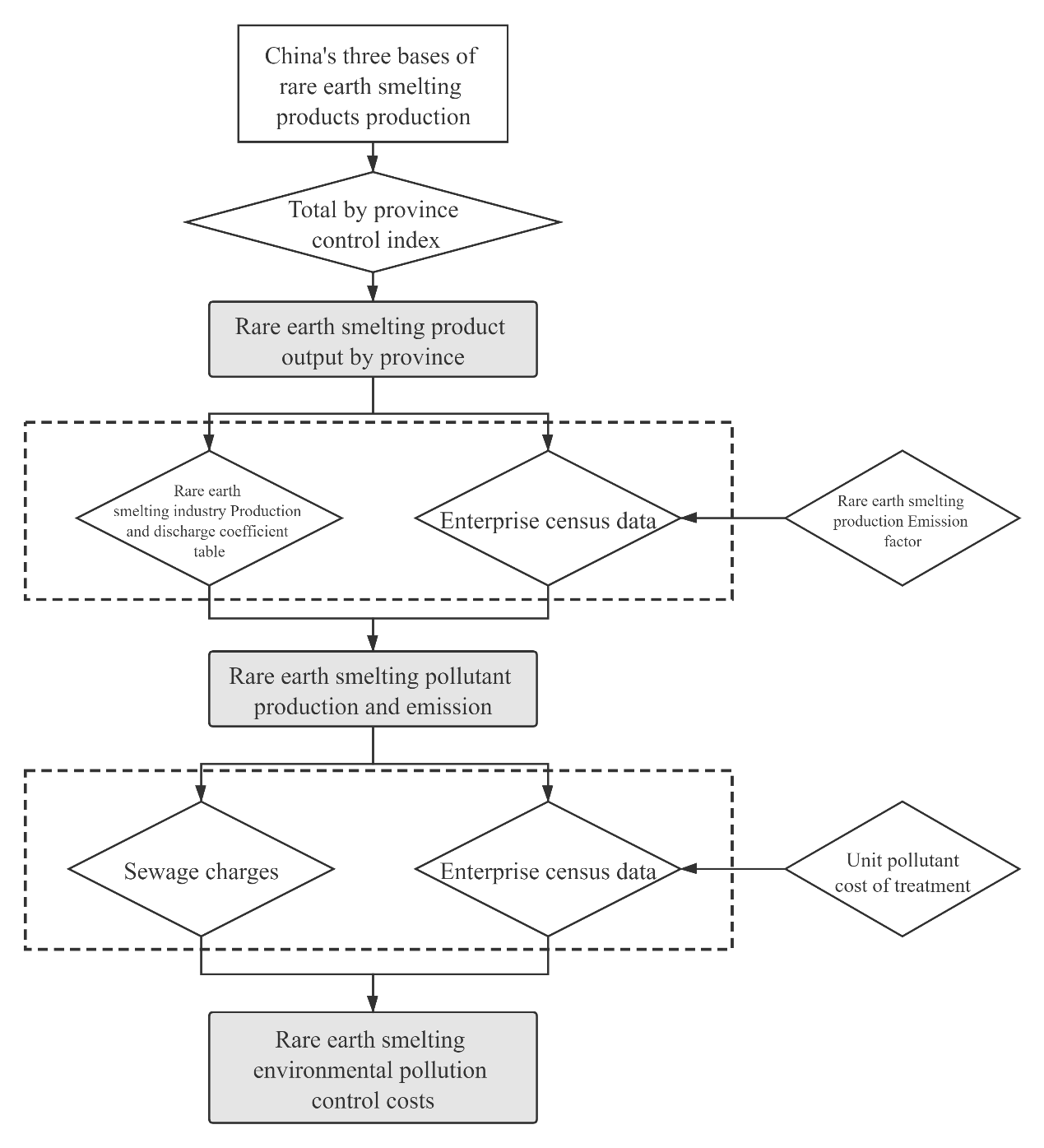
 Fig. S7. Technical route of environmental pollution treatment cost accounting mining

# 7. Weights of the thirty evaluation factors.

Table. S7. Multi-person AHP method results statistics table.

| **System** | **Criteria layer** | **Indicators (units)** | **Jing-Jing Bai** | **Yao-Ting Duan** | **Zhao-Chuo Feng** | **Xu-Hua Li** | **Zhan-Bao Liang** | **Xue-feng Liu** | **Hao Pang** | **Zhen-Bo Sun** | **Jin Wang** | **Xiao-Yan Wu** | **Zi-Yang Xu** | **Xin Xu** | **Qian Zhang** |
| --- | --- | --- | --- | --- | --- | --- | --- | --- | --- | --- | --- | --- | --- | --- | --- |
| Support | Climate Conditions | S_1-1_ | 0.1508 | 0.0285 | 0.1591 | 0.0534 | 0.2079 | 0.3642 | 0.3017 | 0.0361 | 0.1067 | 0.0417 | 0.0940 | 0.0709 | 0.0451 |
|  |  | S_1-2_ | 0.0856 | 0.2018 | 0.0802 | 0.0261 | 0.1597 | 0.0656 | 0.1895 | 0.0470 | 0.0419 | 0.2744 | 0.0402 | 0.0684 | 0.0778 |
|  |  | S_1-3_ | 0.1097 | 0.0730 | 0.1129 | 0.0304 | 0.2999 | 0.1397 | 0.0552 | 0.1602 | 0.0965 | 0.2211 | 0.2188 | 0.0932 | 0.3567 |
|  |  | S_2-1_ | 0.1097 | 0.0235 | 0.2245 | 0.0128 | 0.0643 | 0.1171 | 0.1006 | 0.0699 | 0.2023 | 0.0506 | 0.0931 | 0.1859 | 0.1397 |
|  |  | S_2-2_ | 0.0560 | 0.0686 | 0.0480 | 0.2137 | 0.0213 | 0.0166 | 0.1187 | 0.0725 | 0.1364 | 0.0213 | 0.0982 | 0.0754 | 0.0478 |
|  | Resource Endowment | S_2-3_ | 0.0770 | 0.0759 | 0.1413 | 0.2846 | 0.0337 | 0.0501 | 0.0897 | 0.2874 | 0.0513 | 0.0788 | 0.0300 | 0.1064 | 0.0245 |
|  |  | S_2-4_ | 0.0437 | 0.0281 | 0.0343 | 0.0854 | 0.0319 | 0.0378 | 0.0264 | 0.0474 | 0.1498 | 0.0334 | 0.0215 | 0.0535 | 0.0607 |
|  |  | S_2-5_ | 0.2790 | 0.3930 | 0.0673 | 0.1121 | 0.0912 | 0.0089 | 0.0335 | 0.0885 | 0.0539 | 0.0158 | 0.2963 | 0.2250 | 0.1401 |
|  |  | S_2-6_ | 0.0267 | 0.0552 | 0.0096 | 0.0242 | 0.0139 | 0.0438 | 0.0082 | 0.0635 | 0.0323 | 0.0889 | 0.0132 | 0.0387 | 0.0098 |
|  | Environmental Governance | S_3-1_ | 0.0101 | 0.0146 | 0.0175 | 0.0515 | 0.0172 | 0.0185 | 0.0165 | 0.0307 | 0.0117 | 0.0506 | 0.0106 | 0.0193 | 0.0058 |
|  |  | S_3-2_ | 0.0073 | 0.0189 | 0.0386 | 0.0163 | 0.0113 | 0.0310 | 0.0085 | 0.0468 | 0.0238 | 0.0573 | 0.0431 | 0.0202 | 0.0336 |
|  |  | S_3-3_ | 0.0037 | 0.0074 | 0.0195 | 0.0459 | 0.0342 | 0.0168 | 0.0105 | 0.0118 | 0.0500 | 0.0266 | 0.0089 | 0.0132 | 0.0368 |
|  |  | S_3-4_ | 0.0214 | 0.0054 | 0.0189 | 0.0253 | 0.0059 | 0.0781 | 0.0206 | 0.0204 | 0.0317 | 0.0191 | 0.0221 | 0.0101 | 0.0075 |
|  | Economic Development | S_4-1_ | 0.0186 | 0.0065 | 0.0287 | 0.0178 | 0.0077 | 0.0119 | 0.0210 | 0.0174 | 0.0117 | 0.0208 | 0.0101 | 0.0191 | 0.0147 |
|  |  | S_4-2_ | 0.0307 | 0.0398 | 0.0320 | 0.0899 | 0.1117 | 0.2645 | 0.0325 | 0.0106 | 0.0231 | 0.0446 | 0.0612 | 0.0677 | 0.0208 |
|  |  | S_4-3_ | 0.0307 | 0.0627 | 0.0502 | 0.0223 | 0.1016 | 0.0359 | 0.0196 | 0.0154 | 0.0386 | 0.1351 | 0.2178 | 0.2827 | 0.0614 |
| Pressure | Social pressure | P_1-1_ | 0.0307 | 0.0991 | 0.1183 | 0.0529 | 0.3674 | 0.0840 | 0.0827 | 0.0437 | 0.0668 | 0.4098 | 0.1612 | 0.0801 | 0.0251 |
|  |  | P_1-2_ | 0.0198 | 0.0059 | 0.0514 | 0.0372 | 0.0468 | 0.0167 | 0.0813 | 0.0209 | 0.0308 | 0.0948 | 0.0094 | 0.1106 | 0.0867 |
|  |  | P_1-3_ | 0.0375 | 0.0061 | 0.0756 | 0.0634 | 0.0189 | 0.0036 | 0.0299 | 0.0132 | 0.0273 | 0.0223 | 0.0159 | 0.0396 | 0.0747 |
|  |  | P_1-4_ | 0.0672 | 0.0071 | 0.0944 | 0.0160 | 0.0299 | 0.0071 | 0.0434 | 0.0337 | 0.0538 | 0.0866 | 0.0238 | 0.0691 | 0.0250 |
|  |  | P_2-1_ | 0.0489 | 0.0174 | 0.1195 | 0.0208 | 0.0092 | 0.0038 | 0.0280 | 0.0094 | 0.0204 | 0.0087 | 0.0120 | 0.0214 | 0.0550 |
|  |  | P_2-2_ | 0.0267 | 0.0370 | 0.0339 | 0.0540 | 0.0068 | 0.0178 | 0.0763 | 0.0247 | 0.0181 | 0.0509 | 0.0102 | 0.0111 | 0.0333 |
|  |  | P_2-3_ | 0.0971 | 0.0254 | 0.0814 | 0.0258 | 0.0172 | 0.0083 | 0.1295 | 0.0454 | 0.0594 | 0.0138 | 0.0379 | 0.0381 | 0.0375 |
|  | Social pressure | P_2-4_ | 0.0878 | 0.2753 | 0.0542 | 0.0140 | 0.0511 | 0.0285 | 0.0133 | 0.0517 | 0.0805 | 0.0085 | 0.0288 | 0.0651 | 0.0600 |
|  |  | P_3-1_ | 0.0447 | 0.0669 | 0.1042 | 0.0124 | 0.0895 | 0.1280 | 0.0239 | 0.1130 | 0.0805 | 0.0253 | 0.1209 | 0.0289 | 0.0372 |
|  |  | P_3-2_ | 0.0537 | 0.0670 | 0.0317 | 0.0232 | 0.0222 | 0.2026 | 0.0106 | 0.1223 | 0.0805 | 0.0333 | 0.0575 | 0.0411 | 0.1482 |
|  |  | P_3-3_ | 0.1552 | 0.2119 | 0.0709 | 0.0312 | 0.0653 | 0.0787 | 0.0408 | 0.2332 | 0.2332 | 0.0042 | 0.0653 | 0.0689 | 0.1035 |
|  |  | P_3-4_ | 0.0479 | 0.0047 | 0.0323 | 0.1215 | 0.0238 | 0.0707 | 0.2189 | 0.1046 | 0.0469 | 0.0418 | 0.0214 | 0.0266 | 0.1064 |
|  |  | P_3-5_ | 0.0608 | 0.0573 | 0.0393 | 0.2344 | 0.0315 | 0.0099 | 0.1262 | 0.1284 | 0.1152 | 0.0065 | 0.1171 | 0.0370 | 0.0847 |
|  |  | P_3-6_ | 0.1605 | 0.0166 | 0.0114 | 0.1810 | 0.0069 | 0.0395 | 0.0438 | 0.0296 | 0.0248 | 0.0137 | 0.0388 | 0.0126 | 0.0413 |

Table. S8. Weights of the thirty evaluation factors.

| **System** | **Criteria layer** | **Indicators (units)** | **Weight calculated by the AHP method** | **Weight calculated by the entropy weighting method** | **weight** |
| --- | --- | --- | --- | --- | --- |
| Support | Climate Conditions | S_1-1_ | 0.0604 | 0.0321 | 0.0513 |
|  |  | S_1-2_ | 0.0792 | 0.0166 | 0.0530 |
|  |  | S_1-3_ | 0.0437 | 0.0245 | 0.0391 |
|  |  | S_2-1_ | 0.1213 | 0.0320 | 0.0817 |
|  |  | S_2-2_ | 0.0295 | 0.0413 | 0.0405 |
|  | Resource Endowment | S_2-3_ | 0.0394 | 0.0507 | 0.0501 |
|  |  | S_2-4_ | 0.0254 | 0.1501 | 0.0928 |
|  |  | S_2-5_ | 0.0274 | 0.1241 | 0.0808 |
|  |  | S_2-6_ | 0.0440 | 0.0593 | 0.0567 |
|  | Environmental Governance | S_3-1_ | 0.0596 | 0.0070 | 0.0383 |
|  |  | S_3-2_ | 0.0639 | 0.0112 | 0.0426 |
|  |  | S_3-3_ | 0.0653 | 0.0125 | 0.0440 |
|  |  | S_3-4_ | 0.1014 | 0.0999 | 0.1057 |
|  | Economic Development | S_4-1_ | 0.0633 | 0.0487 | 0.0611 |
|  |  | S_4-2_ | 0.0772 | 0.0676 | 0.0775 |
|  |  | S_4-3_ | 0.0443 | 0.1154 | 0.0849 |
| Pressure | Social pressure | P_1-1_ | 0.1254 | 0.1036 | 0.1173 |
|  |  | P_1-2_ | 0.1022 | 0.0426 | 0.0752 |
|  |  | P_1-3_ | 0.1490 | 0.1172 | 0.1359 |
|  |  | P_1-4_ | 0.1049 | 0.0916 | 0.1010 |
|  |  | P_2-1_ | 0.0742 | 0.0761 | 0.0779 |
|  |  | P_2-2_ | 0.1000 | 0.1509 | 0.1283 |
|  |  | P_2-3_ | 0.0480 | 0.1228 | 0.0882 |
|  | Social pressure | P_2-4_ | 0.1365 | 0.0532 | 0.0976 |
|  |  | P_3-1_ | 0.0306 | 0.0137 | 0.0249 |
|  |  | P_3-2_ | 0.0188 | 0.0156 | 0.0200 |
|  |  | P_3-3_ | 0.0251 | 0.0138 | 0.0223 |
|  |  | P_3-4_ | 0.0196 | 0.0365 | 0.0308 |
|  |  | P_3-5_ | 0.0197 | 0.0420 | 0.0336 |
|  |  | P_3-6_ | 0.0135 | 0.0750 | 0.0470 |

# 8. Primary data of RECC of typical rare earth mining areas in China

Table. S9. Bayan Obo RECC data.

| **System** | **Criteria layer** | **Indicators (units)** | **2019** | **2018** | **2017** | **2016** | **2015** | **2014** | **2013** | **2012** |
| --- | --- | --- | --- | --- | --- | --- | --- | --- | --- | --- |
| Support | Climate Conditions | S_1-1_ | 0.0007 | 0.0000 | 0.0012 | 0.0014 | 0.0016 | 0.0019 | 0.0021 | 0.0023 |
|  |  | S_1-2_ | 0.0087 | 0.0116 | 0.0073 | 0.0058 | 0.0102 | 0.0087 | 0.0087 | 0.0000 |
|  |  | S_1-3_ | 0.0011 | 0.0018 | 0.0024 | 0.0018 | 0.0026 | 0.0026 | 0.0022 | 0.0000 |
|  |  | S_2-1_ | 0.0032 | 0.0061 | 0.0000 | 0.0053 | 0.0004 | 0.0064 | 0.0012 | 0.0083 |
|  |  | S_2-2_ | 0.0181 | 0.0181 | 0.0181 | 0.0182 | 0.0182 | 0.0182 | 0.0181 | 0.0181 |
|  | Resource Endowment | S_2-3_ | 0.0449 | 0.0451 | 0.0417 | 0.0414 | 0.0420 | 0.0397 | 0.0407 | 0.0423 |
|  |  | S_2-4_ | 0.0870 | 0.0871 | 0.0871 | 0.0873 | 0.0874 | 0.0875 | 0.0876 | 0.0877 |
|  |  | S_2-5_ | 0.0000 | 0.0000 | 0.0000 | 0.0000 | 0.0000 | 0.0000 | 0.0000 | 0.0000 |
|  |  | S_2-6_ | 0.0017 | 0.0018 | 0.0017 | 0.0018 | 0.0009 | 0.0014 | 0.0011 | 0.0009 |
|  | Environmental Governance | S_3-1_ | 0.0260 | 0.0251 | 0.0220 | 0.0202 | 0.0333 | 0.0304 | 0.0218 | 0.0216 |
|  |  | S_3-2_ | 0.0360 | 0.0360 | 0.0343 | 0.0324 | 0.0326 | 0.0299 | 0.0298 | 0.0297 |
|  |  | S_3-3_ | 0.0389 | 0.0387 | 0.0371 | 0.0368 | 0.0358 | 0.0347 | 0.0334 | 0.0362 |
|  |  | S_3-4_ | 0.0132 | 0.0084 | 0.0071 | 0.0233 | 0.0260 | 0.0118 | 0.0145 | 0.0084 |
|  | Economic Development | S_4-1_ | 0.0084 | 0.0077 | 0.0063 | 0.0045 | 0.0031 | 0.0019 | 0.0010 | 0.0000 |
|  |  | S_4-2_ | 0.0510 | 0.0724 | 0.0654 | 0.0621 | 0.0601 | 0.0587 | 0.0558 | 0.0567 |
|  |  | S_4-3_ | 0.0799 | 0.0745 | 0.0788 | 0.0769 | 0.0777 | 0.0781 | 0.0780 | 0.0781 |
| Pressure | Social pressure | P_1-1_ | 0.0115 | 0.0114 | 0.0115 | 0.0114 | 0.1145 | 0.0115 | 0.0114 | 0.0050 |
|  |  | P_1-2_ | 0.0724 | 0.0723 | 0.0723 | 0.0722 | 0.0722 | 0.0722 | 0.0722 | 0.0361 |
|  |  | P_1-3_ | 0.0090 | 0.0079 | 0.0090 | 0.0085 | 0.0086 | 0.0078 | 0.0084 | 0.0042 |
|  |  | P_1-4_ | 0.0011 | 0.0011 | 0.0011 | 0.0011 | 0.0011 | 0.0011 | 0.0009 | 0.0005 |
|  |  | P_2-1_ | 0.0751 | 0.0746 | 0.0698 | 0.0729 | 0.0736 | 0.0747 | 0.0627 | 0.0314 |
|  |  | P_2-2_ | 0.1255 | 0.1142 | 0.1189 | 0.1237 | 0.1220 | 0.1253 | 0.1053 | 0.0526 |
|  |  | P_2-3_ | 0.0105 | 0.0099 | 0.0084 | 0.0084 | 0.0081 | 0.0102 | 0.0086 | 0.0043 |
|  | Social pressure | P_2-4_ | 0.0948 | 0.0947 | 0.0945 | 0.0947 | 0.0948 | 0.0948 | 0.0948 | 0.0948 |
|  |  | P_3-1_ | 0.0216 | 0.0199 | 0.0212 | 0.0214 | 0.0213 | 0.0212 | 0.0212 | 0.0212 |
|  |  | P_3-2_ | 0.0151 | 0.0172 | 0.0166 | 0.0167 | 0.0171 | 0.0169 | 0.0171 | 0.0172 |
|  |  | P_3-3_ | 0.0178 | 0.0180 | 0.0182 | 0.0167 | 0.0155 | 0.0173 | 0.0141 | 0.0167 |
|  |  | P_3-4_ | 0.0023 | 0.0023 | 0.0023 | 0.0023 | 0.0023 | 0.0050 | 0.0041 | 0.0035 |
|  |  | P_3-5_ | 0.0233 | 0.0243 | 0.0253 | 0.0278 | 0.0292 | 0.0297 | 0.0300 | 0.0308 |
|  |  | P_3-6_ | 0.0443 | 0.0443 | 0.0443 | 0.0443 | 0.0443 | 0.0443 | 0.0372 | 0.0186 |

Table. S10. Weishan RECC data.

| **System** | **Criteria layer** | **Indicators (units)** | **2019** | **2018** | **2017** | **2016** | **2015** | **2014** | **2013** | **2012** |
| --- | --- | --- | --- | --- | --- | --- | --- | --- | --- | --- |
| Support | Climate Conditions | S_1-1_ | 0.0143 | 0.0143 | 0.0143 | 0.0143 | 0.0143 | 0.0143 | 0.0143 | 0.0143 |
|  |  | S_1-2_ | 0.0203 | 0.0229 | 0.0235 | 0.0276 | 0.0269 | 0.0266 | 0.0264 | 0.0241 |
|  |  | S_1-3_ | 0.0182 | 0.0180 | 0.0180 | 0.0176 | 0.0167 | 0.0174 | 0.0167 | 0.0156 |
|  |  | S_2-1_ | 0.0132 | 0.0225 | 0.0200 | 0.0216 | 0.0172 | 0.0140 | 0.0165 | 0.0123 |
|  |  | S_2-2_ | 0.0354 | 0.0354 | 0.0233 | 0.0233 | 0.0233 | 0.0231 | 0.0231 | 0.0231 |
|  | Resource Endowment | S_2-3_ | 0.0022 | 0.0022 | 0.0022 | 0.0022 | 0.0022 | 0.0021 | 0.0021 | 0.0021 |
|  |  | S_2-4_ | 0.0256 | 0.0256 | 0.0256 | 0.0256 | 0.0256 | 0.0256 | 0.0256 | 0.0256 |
|  |  | S_2-5_ | 0.0000 | 0.0000 | 0.0000 | 0.0000 | 0.0000 | 0.0000 | 0.0000 | 0.0000 |
|  |  | S_2-6_ | 0.0000 | 0.0000 | 0.0035 | 0.0000 | 0.0035 | 0.0035 | 0.0036 | 0.0036 |
|  | Environmental Governance | S_3-1_ | 0.0218 | 0.0214 | 0.0220 | 0.0202 | 0.0333 | 0.0304 | 0.0218 | 0.0216 |
|  |  | S_3-2_ | 0.0370 | 0.0366 | 0.0364 | 0.0361 | 0.0367 | 0.0350 | 0.0343 | 0.0340 |
|  |  | S_3-3_ | 0.0379 | 0.0375 | 0.0371 | 0.0368 | 0.0358 | 0.0347 | 0.0334 | 0.0362 |
|  |  | S_3-4_ | 0.0186 | 0.0223 | 0.0193 | 0.0226 | 0.0191 | 0.0186 | 0.0223 | 0.0206 |
|  | Economic Development | S_4-1_ | 0.0410 | 0.0364 | 0.0307 | 0.0273 | 0.0237 | 0.0194 | 0.0169 | 0.0149 |
|  |  | S_4-2_ | 0.0217 | 0.0299 | 0.0200 | 0.0183 | 0.0166 | 0.0153 | 0.0135 | 0.0116 |
|  |  | S_4-3_ | 0.0038 | 0.0038 | 0.0038 | 0.0038 | 0.0038 | 0.0038 | 0.0038 | 0.0038 |
| Pressure | Social pressure | P_1-1_ | 0.0004 | 0.0004 | 0.0004 | 0.0004 | 0.0004 | 0.0004 | 0.0002 | 0.0000 |
|  |  | P_1-2_ | 0.0031 | 0.0031 | 0.0031 | 0.0031 | 0.0031 | 0.0031 | 0.0018 | 0.0000 |
|  |  | P_1-3_ | 0.0024 | 0.0024 | 0.0024 | 0.0024 | 0.0024 | 0.0024 | 0.0014 | 0.0000 |
|  |  | P_1-4_ | 0.0001 | 0.0001 | 0.0001 | 0.0001 | 0.0001 | 0.0001 | 0.0000 | 0.0000 |
|  |  | P_2-1_ | 0.0044 | 0.0044 | 0.0044 | 0.0044 | 0.0044 | 0.0044 | 0.0025 | 0.0000 |
|  |  | P_2-2_ | 0.0004 | 0.0004 | 0.0004 | 0.0004 | 0.0004 | 0.0004 | 0.0002 | 0.0000 |
|  |  | P_2-3_ | 0.0089 | 0.0089 | 0.0089 | 0.0089 | 0.0089 | 0.0089 | 0.0051 | 0.0000 |
|  | Social pressure | P_2-4_ | 0.0059 | 0.0059 | 0.0059 | 0.0059 | 0.0059 | 0.0059 | 0.0059 | 0.0059 |
|  |  | P_3-1_ | 0.0123 | 0.0123 | 0.0123 | 0.0123 | 0.0123 | 0.0123 | 0.0123 | 0.0133 |
|  |  | P_3-2_ | 0.0024 | 0.0046 | 0.0060 | 0.0061 | 0.0068 | 0.0074 | 0.0077 | 0.0086 |
|  |  | P_3-3_ | 0.0155 | 0.0175 | 0.0195 | 0.0195 | 0.0191 | 0.0193 | 0.0185 | 0.0173 |
|  |  | P_3-4_ | 0.0024 | 0.0000 | 0.0028 | 0.0037 | 0.0040 | 0.0044 | 0.0035 | 0.0037 |
|  |  | P_3-5_ | 0.0005 | 0.0005 | 0.0000 | 0.0007 | 0.0015 | 0.0016 | 0.0025 | 0.0033 |
|  |  | P_3-6_ | 0.0019 | 0.0019 | 0.0019 | 0.0019 | 0.0019 | 0.0019 | 0.0011 | 0.0000 |

Table. S11. Longnan RECC data.

| **System** | **Criteria layer** | **Indicators (units)** | **2019** | **2018** | **2017** | **2016** | **2015** | **2014** | **2013** | **2012** |
| --- | --- | --- | --- | --- | --- | --- | --- | --- | --- | --- |
| Support | Climate Conditions | S_1-1_ | 0.0373 | 0.0385 | 0.0396 | 0.0352 | 0.0324 | 0.0296 | 0.0267 | 0.0239 |
|  |  | S_1-2_ | 0.0406 | 0.0421 | 0.0406 | 0.0421 | 0.0436 | 0.0377 | 0.0348 | 0.0436 |
|  |  | S_1-3_ | 0.0253 | 0.0248 | 0.0257 | 0.0253 | 0.0248 | 0.0255 | 0.0242 | 0.0240 |
|  |  | S_2-1_ | 0.0675 | 0.0645 | 0.0593 | 0.0681 | 0.0738 | 0.0597 | 0.0464 | 0.0767 |
|  |  | S_2-2_ | 0.0146 | 0.0148 | 0.0146 | 0.0148 | 0.0146 | 0.0145 | 0.0143 | 0.0145 |
|  | Resource Endowment | S_2-3_ | 0.0031 | 0.0024 | 0.0022 | 0.0014 | 0.0010 | 0.0005 | 0.0003 | 0.0000 |
|  |  | S_2-4_ | 0.0053 | 0.0053 | 0.0053 | 0.0054 | 0.0054 | 0.0034 | 0.0034 | 0.0054 |
|  |  | S_2-5_ | 0.0757 | 0.0757 | 0.0757 | 0.0757 | 0.0757 | 0.0757 | 0.0757 | 0.0757 |
|  |  | S_2-6_ | 0.0174 | 0.0184 | 0.0170 | 0.0189 | 0.0207 | 0.0171 | 0.0150 | 0.0230 |
|  | Environmental Governance | S_3-1_ | 0.0212 | 0.0191 | 0.0166 | 0.0173 | 0.0261 | 0.0258 | 0.0254 | 0.0248 |
|  |  | S_3-2_ | 0.0369 | 0.0364 | 0.0357 | 0.0324 | 0.0312 | 0.0290 | 0.0286 | 0.0293 |
|  |  | S_3-3_ | 0.0389 | 0.0373 | 0.0367 | 0.0343 | 0.0294 | 0.0276 | 0.0240 | 0.0289 |
|  |  | S_3-4_ | 0.0138 | 0.0125 | 0.0179 | 0.0152 | 0.0125 | 0.0111 | 0.0003 | 0.0003 |
|  | Economic Development | S_4-1_ | 0.0464 | 0.0456 | 0.0317 | 0.0225 | 0.0121 | 0.0117 | 0.0108 | 0.0077 |
|  |  | S_4-2_ | 0.0163 | 0.0179 | 0.0136 | 0.0119 | 0.0102 | 0.0091 | 0.0077 | 0.0060 |
|  |  | S_4-3_ | 0.0451 | 0.0451 | 0.0451 | 0.0451 | 0.0451 | 0.0451 | 0.0451 | 0.0451 |
| Pressure | Social pressure | P_1-1_ | 0.0072 | 0.0072 | 0.0072 | 0.0072 | 0.0064 | 0.0072 | 0.0072 | 0.0036 |
|  |  | P_1-2_ | 0.0524 | 0.0524 | 0.0524 | 0.0524 | 0.0464 | 0.0524 | 0.0524 | 0.0262 |
|  |  | P_1-3_ | 0.1331 | 0.1331 | 0.1331 | 0.1331 | 0.1178 | 0.1331 | 0.1331 | 0.0666 |
|  |  | P_1-4_ | 0.0983 | 0.0983 | 0.0983 | 0.0983 | 0.0869 | 0.0983 | 0.0983 | 0.0491 |
|  |  | P_2-1_ | 0.0085 | 0.0085 | 0.0085 | 0.0085 | 0.0075 | 0.0085 | 0.0085 | 0.0043 |
|  |  | P_2-2_ | 0.0010 | 0.0010 | 0.0010 | 0.0010 | 0.0009 | 0.0010 | 0.0010 | 0.0005 |
|  |  | P_2-3_ | 0.0001 | 0.0001 | 0.0001 | 0.0001 | 0.0001 | 0.0001 | 0.0001 | 0.0000 |
|  | Social pressure | P_2-4_ | 0.0598 | 0.0598 | 0.0598 | 0.0598 | 0.0598 | 0.0598 | 0.0598 | 0.0598 |
|  |  | P_3-1_ | 0.0158 | 0.0158 | 0.0158 | 0.0159 | 0.0159 | 0.0151 | 0.0141 | 0.0123 |
|  |  | P_3-2_ | 0.0085 | 0.0085 | 0.0092 | 0.0092 | 0.0096 | 0.0103 | 0.0103 | 0.0097 |
|  |  | P_3-3_ | 0.0042 | 0.0041 | 0.0040 | 0.0037 | 0.0035 | 0.0033 | 0.0031 | 0.0029 |
|  |  | P_3-4_ | 0.0036 | 0.0040 | 0.0041 | 0.0040 | 0.0039 | 0.0039 | 0.0039 | 0.0040 |
|  |  | P_3-5_ | 0.0028 | 0.0020 | 0.0025 | 0.0030 | 0.0040 | 0.0044 | 0.0048 | 0.0052 |
|  |  | P_3-6_ | 0.0067 | 0.0067 | 0.0067 | 0.0067 | 0.0059 | 0.0067 | 0.0067 | 0.0033 |

Table. S12. Mianning RECC data.

| **System** | **Criteria layer** | **Indicators (units)** | **2019** | **2018** | **2017** | **2016** | **2015** | **2014** | **2013** | **2012** |
| --- | --- | --- | --- | --- | --- | --- | --- | --- | --- | --- |
| Support | Climate Conditions | S_1-1_ | 0.0319 | 0.0347 | 0.0368 | 0.0352 | 0.0364 | 0.0352 | 0.0357 | 0.0338 |
|  |  | S_1-2_ | 0.0465 | 0.0421 | 0.0436 | 0.0450 | 0.0421 | 0.0450 | 0.0392 | 0.0465 |
|  |  | S_1-3_ | 0.0240 | 0.0226 | 0.0242 | 0.0218 | 0.0222 | 0.0200 | 0.0226 | 0.0191 |
|  |  | S_2-1_ | 0.0481 | 0.0511 | 0.0297 | 0.0531 | 0.0457 | 0.0526 | 0.0555 | 0.0593 |
|  |  | S_2-2_ | 0.0093 | 0.0093 | 0.0093 | 0.0093 | 0.0094 | 0.0036 | 0.0020 | 0.0036 |
|  | Resource Endowment | S_2-3_ | 0.0306 | 0.0276 | 0.0258 | 0.0252 | 0.0251 | 0.0241 | 0.0231 | 0.0215 |
|  |  | S_2-4_ | 0.0037 | 0.0037 | 0.0038 | 0.0030 | 0.0039 | 0.0039 | 0.0040 | 0.0040 |
|  |  | S_2-5_ | 0.0000 | 0.0000 | 0.0000 | 0.0000 | 0.0000 | 0.0000 | 0.0000 | 0.0000 |
|  |  | S_2-6_ | 0.0364 | 0.0355 | 0.0347 | 0.0358 | 0.0369 | 0.0359 | 0.0368 | 0.0517 |
|  | Environmental Governance | S_3-1_ | 0.0196 | 0.0189 | 0.0175 | 0.0172 | 0.0200 | 0.0192 | 0.0184 | 0.0206 |
|  |  | S_3-2_ | 0.0249 | 0.0235 | 0.0226 | 0.0194 | 0.0317 | 0.0299 | 0.0286 | 0.0289 |
|  |  | S_3-3_ | 0.0300 | 0.0289 | 0.0276 | 0.0232 | 0.0364 | 0.0364 | 0.0355 | 0.0339 |
|  |  | S_3-4_ | 0.0599 | 0.0938 | 0.1006 | 0.0959 | 0.0803 | 0.0674 | 0.0593 | 0.0498 |
|  | Economic Development | S_4-1_ | 0.0280 | 0.0214 | 0.0158 | 0.0139 | 0.0113 | 0.0060 | 0.0047 | 0.0040 |
|  |  | S_4-2_ | 0.0090 | 0.0076 | 0.0070 | 0.0064 | 0.0057 | 0.0048 | 0.0038 | 0.0024 |
|  |  | S_4-3_ | 0.0055 | 0.0055 | 0.0055 | 0.0055 | 0.0055 | 0.0055 | 0.0055 | 0.0055 |
| Pressure | Social pressure | P_1-1_ | 0.0040 | 0.0040 | 0.0040 | 0.0040 | 0.0040 | 0.0040 | 0.0039 | 0.0000 |
|  |  | P_1-2_ | 0.0293 | 0.0293 | 0.0293 | 0.0293 | 0.0293 | 0.0293 | 0.0286 | 0.0000 |
|  |  | P_1-3_ | 0.0106 | 0.0106 | 0.0106 | 0.0106 | 0.0106 | 0.0106 | 0.0103 | 0.0000 |
|  |  | P_1-4_ | 0.0167 | 0.0167 | 0.0167 | 0.0167 | 0.0167 | 0.0167 | 0.0163 | 0.0000 |
|  | Social pressure |  | 0.0422 | 0.0422 | 0.0422 | 0.0422 | 0.0422 | 0.0422 | 0.0412 | 0.0000 |
|  |  | P_2-1_ | 0.0034 | 0.0034 | 0.0034 | 0.0034 | 0.0034 | 0.0034 | 0.0033 | 0.0000 |
|  |  | P_2-2_ | 0.0854 | 0.0854 | 0.0854 | 0.0854 | 0.0854 | 0.0854 | 0.0834 | 0.0000 |
|  |  | P_2-3_ | 0.0000 | 0.0000 | 0.0000 | 0.0000 | 0.0000 | 0.0000 | 0.0000 | 0.0000 |
|  | Social pressure | P_2-4_ | 0.0215 | 0.0199 | 0.0203 | 0.0197 | 0.0222 | 0.0215 | 0.0215 | 0.0215 |
|  |  |  | 0.0045 | 0.0084 | 0.0084 | 0.0096 | 0.0090 | 0.0096 | 0.0095 | 0.0086 |
|  |  | P_3-1_ | 0.0127 | 0.0132 | 0.0125 | 0.0133 | 0.0126 | 0.0134 | 0.0119 | 0.0121 |
|  |  | P_3-2_ | 0.0047 | 0.0045 | 0.0065 | 0.0064 | 0.0206 | 0.0206 | 0.0281 | 0.0275 |
|  |  | P_3-3_ | 0.0056 | 0.0058 | 0.0053 | 0.0059 | 0.0062 | 0.0067 | 0.0075 | 0.0069 |

Table. S13. Pingyuan RECC data.

| **System** | **Criteria layer** | **Indicators (units)** | **2019** | **2018** | **2017** | **2016** | **2015** | **2014** | **2013** | **2012** |
| --- | --- | --- | --- | --- | --- | --- | --- | --- | --- | --- |
| Support | Climate Conditions | S_1-1_ | 0.0436 | 0.0415 | 0.0427 | 0.0411 | 0.0434 | 0.0408 | 0.0380 | 0.0418 |
|  |  | S_1-2_ | 0.0409 | 0.0412 | 0.0421 | 0.0406 | 0.0392 | 0.0436 | 0.0377 | 0.0421 |
|  |  | S_1-3_ | 0.0290 | 0.0297 | 0.0341 | 0.0334 | 0.0316 | 0.0325 | 0.0321 | 0.0314 |
|  |  | S_2-1_ | 0.0571 | 0.0549 | 0.0586 | 0.0339 | 0.0470 | 0.0339 | 0.0374 | 0.0578 |
|  |  | S_2-2_ | 0.0190 | 0.0190 | 0.0190 | 0.0190 | 0.0190 | 0.0190 | 0.0190 | 0.0190 |
|  | Resource Endowment | S_2-3_ | 0.0370 | 0.0370 | 0.0367 | 0.0367 | 0.0366 | 0.0366 | 0.0365 | 0.0365 |
|  |  | S_2-4_ | 0.0001 | 0.0001 | 0.0001 | 0.0001 | 0.0001 | 0.0001 | 0.0001 | 0.0001 |
|  |  | S_2-5_ | 0.0258 | 0.0258 | 0.0258 | 0.0258 | 0.0258 | 0.0258 | 0.0258 | 0.0258 |
|  |  | S_2-6_ | 0.0100 | 0.0100 | 0.0100 | 0.0098 | 0.0095 | 0.0097 | 0.0102 | 0.0096 |
|  | Environmental Governance | S_3-1_ | 0.0196 | 0.0189 | 0.0175 | 0.0172 | 0.0200 | 0.0192 | 0.0184 | 0.0206 |
|  |  | S_3-2_ | 0.0375 | 0.0369 | 0.0366 | 0.0353 | 0.0330 | 0.0307 | 0.0264 | 0.0208 |
|  |  | S_3-3_ | 0.0389 | 0.0389 | 0.0389 | 0.0389 | 0.0389 | 0.0389 | 0.0389 | 0.0389 |
|  |  | S_3-4_ | 0.0050 | 0.0037 | 0.0023 | 0.0030 | 0.0023 | 0.0030 | 0.0023 | 0.0030 |
|  | Economic Development | S_4-1_ | 0.0515 | 0.0498 | 0.0466 | 0.0394 | 0.0327 | 0.0236 | 0.0187 | 0.0113 |
|  |  | S_4-2_ | 0.0048 | 0.0042 | 0.0038 | 0.0035 | 0.0025 | 0.0016 | 0.0006 | 0.0000 |
|  |  | S_4-3_ | 0.0048 | 0.0048 | 0.0048 | 0.0048 | 0.0048 | 0.0048 | 0.0048 | 0.0048 |
| Pressure | Social pressure | P_1-1_ | 0.0041 | 0.0041 | 0.0041 | 0.0041 | 0.0041 | 0.0041 | 0.0041 | 0.0022 |
|  |  | P_1-2_ | 0.0295 | 0.0295 | 0.0295 | 0.0295 | 0.0295 | 0.0295 | 0.0295 | 0.0161 |
|  |  | P_1-3_ | 0.0007 | 0.0007 | 0.0007 | 0.0007 | 0.0007 | 0.0007 | 0.0007 | 0.0004 |
|  |  | P_1-4_ | 0.0227 | 0.0227 | 0.0227 | 0.0227 | 0.0227 | 0.0227 | 0.0227 | 0.0124 |
|  | Social pressure |  | 0.0011 | 0.0011 | 0.0011 | 0.0011 | 0.0011 | 0.0011 | 0.0011 | 0.0006 |
|  |  | P_2-1_ | 0.0001 | 0.0001 | 0.0001 | 0.0001 | 0.0001 | 0.0001 | 0.0001 | 0.0001 |
|  |  | P_2-2_ | 0.0000 | 0.0000 | 0.0000 | 0.0000 | 0.0000 | 0.0000 | 0.0000 | 0.0000 |
|  |  | P_2-3_ | 0.0433 | 0.0433 | 0.0433 | 0.0433 | 0.0433 | 0.0433 | 0.0433 | 0.0433 |
|  | Social pressure | P_2-4_ | 0.0000 | 0.0031 | 0.0041 | 0.0041 | 0.0045 | 0.0054 | 0.0064 | 0.0078 |
|  |  |  | 0.0000 | 0.0003 | 0.0010 | 0.0032 | 0.0028 | 0.0044 | 0.0047 | 0.0058 |
|  |  | P_3-1_ | 0.0086 | 0.0050 | 0.0045 | 0.0046 | 0.0081 | 0.0036 | 0.0099 | 0.0109 |
|  |  | P_3-2_ | 0.0037 | 0.0036 | 0.0039 | 0.0039 | 0.0037 | 0.0037 | 0.0036 | 0.0036 |
|  |  | P_3-3_ | 0.0043 | 0.0040 | 0.0037 | 0.0038 | 0.0041 | 0.0045 | 0.0049 | 0.0071 |

Table. S14. Chongzuo RECC data.

| **System** | **Criteria layer** | **Indicators (units)** | **2019** | **2018** | **2017** | **2016** | **2015** | **2014** | **2013** | **2012** |
| --- | --- | --- | --- | --- | --- | --- | --- | --- | --- | --- |
| Support | Climate Conditions | S_1-1_ | 0.0462 | 0.0462 | 0.0462 | 0.0450 | 0.0455 | 0.0457 | 0.0460 | 0.0453 |
|  |  | S_1-2_ | 0.0472 | 0.0469 | 0.0479 | 0.0465 | 0.0436 | 0.0392 | 0.0465 | 0.0450 |
|  |  | S_1-3_ | 0.0327 | 0.0323 | 0.0324 | 0.0332 | 0.0327 | 0.0327 | 0.0330 | 0.0325 |
|  |  | S_2-1_ | 0.0464 | 0.0444 | 0.0477 | 0.0391 | 0.0431 | 0.0486 | 0.0464 | 0.0408 |
|  |  | S_2-2_ | 0.0000 | 0.0000 | 0.0000 | 0.0000 | 0.0000 | 0.0000 | 0.0000 | 0.0000 |
|  | Resource Endowment | S_2-3_ | 0.0247 | 0.0246 | 0.0245 | 0.0243 | 0.0243 | 0.0243 | 0.0241 | 0.0238 |
|  |  | S_2-4_ | 0.0000 | 0.0000 | 0.0000 | 0.0000 | 0.0000 | 0.0000 | 0.0000 | 0.0000 |
|  |  | S_2-5_ | 0.0493 | 0.0493 | 0.0493 | 0.0215 | 0.0376 | 0.0188 | 0.0254 | 0.0334 |
|  |  | S_2-6_ | 0.0254 | 0.0254 | 0.0254 | 0.0187 | 0.0236 | 0.0298 | 0.0268 | 0.0267 |
|  | Environmental Governance | S_3-1_ | 0.0214 | 0.0223 | 0.0215 | 0.0181 | 0.0162 | 0.0167 | 0.0061 | 0.0000 |
|  |  | S_3-2_ | 0.0325 | 0.0356 | 0.0350 | 0.0000 | 0.0034 | 0.0042 | 0.0096 | 0.0098 |
|  |  | S_3-3_ | 0.0388 | 0.0389 | 0.0389 | 0.0047 | 0.0038 | 0.0000 | 0.0034 | 0.0056 |
|  |  | S_3-4_ | 0.0003 | 0.0000 | 0.0009 | 0.0016 | 0.0030 | 0.0016 | 0.0023 | 0.0009 |
|  | Economic Development | S_4-1_ | 0.0560 | 0.0508 | 0.0185 | 0.0078 | 0.0057 | 0.0053 | 0.0048 | 0.0037 |
|  |  | S_4-2_ | 0.0259 | 0.0246 | 0.0206 | 0.0156 | 0.0136 | 0.0129 | 0.0113 | 0.0099 |
|  |  | S_4-3_ | 0.0000 | 0.0000 | 0.0000 | 0.0000 | 0.0000 | 0.0000 | 0.0000 | 0.0000 |
| Pressure | Social pressure | P_1-1_ | 0.0000 | 0.0000 | 0.0000 | 0.0000 | 0.0000 | 0.0000 | 0.0000 | 0.0000 |
|  |  | P_1-2_ | 0.0002 | 0.0002 | 0.0002 | 0.0002 | 0.0002 | 0.0001 | 0.0002 | 0.0002 |
|  |  | P_1-3_ | 0.0001 | 0.0001 | 0.0001 | 0.0001 | 0.0001 | 0.0001 | 0.0001 | 0.0002 |
|  |  | P_1-4_ | 0.0001 | 0.0001 | 0.0001 | 0.0001 | 0.0001 | 0.0001 | 0.0001 | 0.0001 |
|  | Social pressure |  | 0.0005 | 0.0005 | 0.0005 | 0.0005 | 0.0005 | 0.0003 | 0.0005 | 0.0006 |
|  |  | P_2-1_ | 0.0001 | 0.0001 | 0.0001 | 0.0001 | 0.0001 | 0.0000 | 0.0001 | 0.0001 |
|  |  | P_2-2_ | 0.0000 | 0.0000 | 0.0000 | 0.0000 | 0.0000 | 0.0000 | 0.0000 | 0.0000 |
|  |  | P_2-3_ | 0.0039 | 0.0039 | 0.0039 | 0.0039 | 0.0039 | 0.0039 | 0.0039 | 0.0039 |
|  | Social pressure | P_2-4_ | 0.0035 | 0.0056 | 0.0035 | 0.0121 | 0.0085 | 0.0075 | 0.0022 | 0.0082 |
|  |  |  | 0.0065 | 0.0067 | 0.0068 | 0.0055 | 0.0041 | 0.0050 | 0.0049 | 0.0043 |
|  |  | P_3-1_ | 0.0101 | 0.0086 | 0.0094 | 0.0101 | 0.0000 | 0.0091 | 0.0089 | 0.0085 |
|  |  | P_3-2_ | 0.0024 | 0.0025 | 0.0025 | 0.0025 | 0.0026 | 0.0025 | 0.0025 | 0.0026 |
|  |  | P_3-3_ | 0.0051 | 0.0056 | 0.0058 | 0.0064 | 0.0067 | 0.0077 | 0.0080 | 0.0084 |

# 9. Analysis data of the obstacles of typical rare earth mining areas in China

Table. S15. Bayan Obo obstacle degree analysis data.

| **System** | **Criteria layer** | **Indicators (units)** | **2019** | **2018** | **2017** | **2016** | **2015** | **2014** | **2013** | **2012** |
| --- | --- | --- | --- | --- | --- | --- | --- | --- | --- | --- |
| Support | Climate Conditions | S_1-1_ | 0.0870 | 0.0906 | 0.0850 | 0.0858 | 0.0903 | 0.0874 | 0.0843 | 0.0822 |
|  |  | S_1-2_ | 0.0761 | 0.0731 | 0.0775 | 0.0812 | 0.0769 | 0.0773 | 0.0749 | 0.0896 |
|  |  | S_1-3_ | 0.0654 | 0.0660 | 0.0623 | 0.0643 | 0.0644 | 0.0620 | 0.0609 | 0.0644 |
|  |  | S_2-1_ | 0.1352 | 0.1338 | 0.1387 | 0.1315 | 0.1528 | 0.1385 | 0.1442 | 0.1268 |
|  |  | S_2-2_ | 0.0384 | 0.0395 | 0.0379 | 0.0384 | 0.0364 | 0.0339 | 0.0332 | 0.0339 |
|  | Resource Endowment | S_2-3_ | 0.0089 | 0.0089 | 0.0143 | 0.0150 | 0.0085 | 0.0107 | 0.0083 | 0.0073 |
|  |  | S_2-4_ | 0.0099 | 0.0100 | 0.0096 | 0.0095 | 0.0032 | 0.0004 | 0.0002 | 0.0023 |
|  |  | S_2-5_ | 0.1390 | 0.1428 | 0.1370 | 0.1391 | 0.1518 | 0.1493 | 0.1447 | 0.1402 |
|  |  | S_2-6_ | 0.0947 | 0.0972 | 0.0933 | 0.0946 | 0.1025 | 0.0992 | 0.0967 | 0.0948 |
|  | Environmental Governance | S_3-1_ | 0.0211 | 0.0234 | 0.0277 | 0.0312 | 0.0025 | 0.0056 | 0.0219 | 0.0235 |
|  |  | S_3-2_ | 0.0113 | 0.0116 | 0.0141 | 0.0176 | 0.0122 | 0.0151 | 0.0149 | 0.0166 |
|  |  | S_3-3_ | 0.0087 | 0.0094 | 0.0117 | 0.0124 | 0.0088 | 0.0083 | 0.0105 | 0.0074 |
|  |  | S_3-4_ | 0.1592 | 0.1719 | 0.1673 | 0.1418 | 0.1495 | 0.1751 | 0.1646 | 0.1703 |
|  | Economic Development | S_4-1_ | 0.0906 | 0.0944 | 0.0929 | 0.0974 | 0.1068 | 0.1067 | 0.1052 | 0.1043 |
|  |  | S_4-2_ | 0.0456 | 0.0089 | 0.0205 | 0.0264 | 0.0266 | 0.0271 | 0.0318 | 0.0309 |
|  |  | S_4-3_ | 0.0087 | 0.0185 | 0.0104 | 0.0138 | 0.0069 | 0.0035 | 0.0036 | 0.0056 |

Table. S16. Weishan obstacle degree analysis data.

| **System** | **Criteria layer** | **Indicators (units)** | **2019** | **2018** | **2017** | **2016** | **2015** | **2014** | **2013** | **2012** |
| --- | --- | --- | --- | --- | --- | --- | --- | --- | --- | --- |
| Support | Climate Conditions | S_1-1_ | 0.0537 | 0.0551 | 0.0528 | 0.0526 | 0.0518 | 0.0502 | 0.0495 | 0.0491 |
|  |  | S_1-2_ | 0.0474 | 0.0447 | 0.0420 | 0.0361 | 0.0348 | 0.0336 | 0.0333 | 0.0371 |
|  |  | S_1-3_ | 0.0303 | 0.0314 | 0.0301 | 0.0307 | 0.0291 | 0.0263 | 0.0269 | 0.0292 |
|  |  | S_2-1_ | 0.0994 | 0.0883 | 0.0882 | 0.0856 | 0.0948 | 0.0987 | 0.0934 | 0.0971 |
|  |  | S_2-2_ | 0.0073 | 0.0075 | 0.0245 | 0.0244 | 0.0208 | 0.0194 | 0.0191 | 0.0201 |
|  | Resource Endowment | S_2-3_ | 0.0696 | 0.0714 | 0.0684 | 0.0682 | 0.0689 | 0.0676 | 0.0666 | 0.0654 |
|  |  | S_2-4_ | 0.0976 | 0.1001 | 0.0960 | 0.0956 | 0.0990 | 0.0978 | 0.0964 | 0.0938 |
|  |  | S_2-5_ | 0.1173 | 0.1203 | 0.1154 | 0.1150 | 0.1202 | 0.1192 | 0.1175 | 0.1139 |
|  |  | S_2-6_ | 0.0823 | 0.0845 | 0.0760 | 0.0807 | 0.0771 | 0.0758 | 0.0746 | 0.0730 |
|  | Environmental Governance | S_3-1_ | 0.0240 | 0.0252 | 0.0233 | 0.0258 | 0.0020 | 0.0045 | 0.0178 | 0.0191 |
|  |  | S_3-2_ | 0.0081 | 0.0090 | 0.0088 | 0.0092 | 0.0033 | 0.0041 | 0.0050 | 0.0071 |
|  |  | S_3-3_ | 0.0089 | 0.0097 | 0.0099 | 0.0103 | 0.0069 | 0.0066 | 0.0085 | 0.0060 |
|  |  | S_3-4_ | 0.1263 | 0.1243 | 0.1234 | 0.1182 | 0.1292 | 0.1290 | 0.1215 | 0.1202 |
|  | Economic Development | S_4-1_ | 0.0292 | 0.0368 | 0.0434 | 0.0481 | 0.0524 | 0.0576 | 0.0607 | 0.0627 |
|  |  | S_4-2_ | 0.0810 | 0.0708 | 0.0820 | 0.0842 | 0.0891 | 0.0898 | 0.0913 | 0.0918 |
|  |  | S_4-3_ | 0.1177 | 0.1208 | 0.1158 | 0.1154 | 0.1206 | 0.1197 | 0.1179 | 0.1144 |

Table. S17. Longnan obstacle degree analysis data.

| **System** | **Criteria layer** | **Indicators (units)** | **2019** | **2018** | **2017** | **2016** | **2015** | **2014** | **2013** | **2012** |
| --- | --- | --- | --- | --- | --- | --- | --- | --- | --- | --- |
| Support | Climate Conditions | S_1-1_ | 0.0283 | 0.0256 | 0.0222 | 0.0301 | 0.0314 | 0.0336 | 0.0363 | 0.0461 |
|  |  | S_1-2_ | 0.0249 | 0.0217 | 0.0236 | 0.0203 | 0.0117 | 0.0205 | 0.0244 | 0.0110 |
|  |  | S_1-3_ | 0.0280 | 0.0286 | 0.0257 | 0.0259 | 0.0218 | 0.0173 | 0.0184 | 0.0223 |
|  |  | S_2-1_ | 0.0288 | 0.0345 | 0.0430 | 0.0255 | 0.0086 | 0.0342 | 0.0564 | 0.0025 |
|  |  | S_2-2_ | 0.0522 | 0.0514 | 0.0494 | 0.0480 | 0.0458 | 0.0422 | 0.0393 | 0.0435 |
|  | Resource Endowment | S_2-3_ | 0.0951 | 0.0955 | 0.0918 | 0.0911 | 0.0944 | 0.0898 | 0.0835 | 0.0908 |
|  |  | S_2-4_ | 0.1769 | 0.1751 | 0.1674 | 0.1636 | 0.1739 | 0.1701 | 0.1572 | 0.1637 |
|  |  | S_2-5_ | 0.0102 | 0.0101 | 0.0097 | 0.0095 | 0.0026 | 0.0000 | 0.0000 | 0.0025 |
|  |  | S_2-6_ | 0.0795 | 0.0766 | 0.0759 | 0.0708 | 0.0669 | 0.0697 | 0.0684 | 0.0586 |
|  | Environmental Governance | S_3-1_ | 0.0347 | 0.0384 | 0.0416 | 0.0393 | 0.0176 | 0.0150 | 0.0146 | 0.0190 |
|  |  | S_3-2_ | 0.0115 | 0.0125 | 0.0131 | 0.0191 | 0.0157 | 0.0173 | 0.0168 | 0.0187 |
|  |  | S_3-3_ | 0.0102 | 0.0135 | 0.0139 | 0.0181 | 0.0224 | 0.0229 | 0.0279 | 0.0222 |
|  |  | S_3-4_ | 0.1858 | 0.1866 | 0.1680 | 0.1693 | 0.1860 | 0.1804 | 0.1871 | 0.1990 |
|  | Economic Development | S_4-1_ | 0.0296 | 0.0311 | 0.0562 | 0.0722 | 0.0940 | 0.0894 | 0.0843 | 0.0972 |
|  |  | S_4-2_ | 0.1237 | 0.1192 | 0.1222 | 0.1226 | 0.1320 | 0.1276 | 0.1207 | 0.1325 |
|  |  | S_4-3_ | 0.0805 | 0.0797 | 0.0762 | 0.0745 | 0.0749 | 0.0700 | 0.0648 | 0.0705 |

Table. S18. Mianning obstacle degree analysis data.

| **System** | **Criteria layer** | **Indicators (units)** | **2019** | **2018** | **2017** | **2016** | **2015** | **2014** | **2013** | **2012** |
| --- | --- | --- | --- | --- | --- | --- | --- | --- | --- | --- |
| Support | Climate Conditions | S_1-1_ | 0.0327 | 0.0289 | 0.0242 | 0.0272 | 0.0211 | 0.0208 | 0.0194 | 0.0247 |
|  |  | S_1-2_ | 0.0110 | 0.0189 | 0.0158 | 0.0135 | 0.0134 | 0.0055 | 0.0160 | 0.0049 |
|  |  | S_1-3_ | 0.0256 | 0.0287 | 0.0251 | 0.0294 | 0.0249 | 0.0266 | 0.0210 | 0.0292 |
|  |  | S_2-1_ | 0.0567 | 0.0534 | 0.0874 | 0.0485 | 0.0612 | 0.0455 | 0.0389 | 0.0336 |
|  |  | S_2-2_ | 0.0525 | 0.0543 | 0.0523 | 0.0528 | 0.0518 | 0.0600 | 0.0613 | 0.0597 |
|  | Resource Endowment | S_2-3_ | 0.0330 | 0.0393 | 0.0409 | 0.0422 | 0.0402 | 0.0396 | 0.0404 | 0.0448 |
|  |  | S_2-4_ | 0.1504 | 0.1553 | 0.1495 | 0.1521 | 0.1616 | 0.1583 | 0.1538 | 0.1531 |
|  |  | S_2-5_ | 0.1363 | 0.1408 | 0.1356 | 0.1369 | 0.1461 | 0.1430 | 0.1390 | 0.1388 |
|  |  | S_2-6_ | 0.0342 | 0.0370 | 0.0369 | 0.0354 | 0.0303 | 0.0297 | 0.0274 | 0.0023 |
|  | Environmental Governance | S_3-1_ | 0.0315 | 0.0338 | 0.0349 | 0.0358 | 0.0276 | 0.0265 | 0.0273 | 0.0250 |
|  |  | S_3-2_ | 0.0299 | 0.0332 | 0.0335 | 0.0393 | 0.0135 | 0.0145 | 0.0164 | 0.0179 |
|  |  | S_3-3_ | 0.0237 | 0.0262 | 0.0276 | 0.0352 | 0.0072 | 0.0048 | 0.0064 | 0.0114 |
|  |  | S_3-4_ | 0.0772 | 0.0206 | 0.0085 | 0.0166 | 0.0410 | 0.0627 | 0.0759 | 0.0939 |
|  | Economic Development | S_4-1_ | 0.0558 | 0.0692 | 0.0760 | 0.0799 | 0.0874 | 0.0944 | 0.0943 | 0.0960 |
|  |  | S_4-2_ | 0.1155 | 0.1218 | 0.1184 | 0.1205 | 0.1290 | 0.1277 | 0.1259 | 0.1284 |
|  |  | S_4-3_ | 0.1341 | 0.1385 | 0.1334 | 0.1347 | 0.1436 | 0.1405 | 0.1366 | 0.1364 |

Table. S19. Pingyuan obstacle degree analysis data.

| **System** | **Criteria layer** | **Indicators (units)** | **2019** | **2018** | **2017** | **2016** | **2015** | **2014** | **2013** | **2012** |
| --- | --- | --- | --- | --- | --- | --- | --- | --- | --- | --- |
| Support | Climate Conditions | S_1-1_ | 0.0133 | 0.0167 | 0.0148 | 0.0165 | 0.0074 | 0.0097 | 0.0143 | 0.0099 |
|  |  | S_1-2_ | 0.0209 | 0.0201 | 0.0187 | 0.0199 | 0.0180 | 0.0078 | 0.0178 | 0.0123 |
|  |  | S_1-3_ | 0.0176 | 0.0162 | 0.0087 | 0.0093 | 0.0067 | 0.0028 | 0.0035 | 0.0068 |
|  |  | S_2-1_ | 0.0428 | 0.0461 | 0.0398 | 0.0775 | 0.0559 | 0.0771 | 0.0686 | 0.0350 |
|  |  | S_2-2_ | 0.0373 | 0.0368 | 0.0370 | 0.0348 | 0.0320 | 0.0296 | 0.0287 | 0.0307 |
|  | Resource Endowment | S_2-3_ | 0.0227 | 0.0224 | 0.0232 | 0.0218 | 0.0177 | 0.0153 | 0.0151 | 0.0172 |
|  |  | S_2-4_ | 0.1611 | 0.1588 | 0.1597 | 0.1501 | 0.1608 | 0.1578 | 0.1531 | 0.1543 |
|  |  | S_2-5_ | 0.0957 | 0.0943 | 0.0948 | 0.0891 | 0.0927 | 0.0900 | 0.0873 | 0.0890 |
|  |  | S_2-6_ | 0.0813 | 0.0801 | 0.0806 | 0.0760 | 0.0786 | 0.0756 | 0.0726 | 0.0753 |
|  | Environmental Governance | S_3-1_ | 0.0325 | 0.0332 | 0.0358 | 0.0342 | 0.0263 | 0.0252 | 0.0260 | 0.0241 |
|  |  | S_3-2_ | 0.0088 | 0.0098 | 0.0103 | 0.0118 | 0.0104 | 0.0123 | 0.0195 | 0.0313 |
|  |  | S_3-3_ | 0.0088 | 0.0087 | 0.0087 | 0.0082 | 0.0023 | 0.0000 | 0.0000 | 0.0022 |
|  |  | S_3-4_ | 0.1750 | 0.1747 | 0.1781 | 0.1663 | 0.1801 | 0.1758 | 0.1718 | 0.1717 |
|  | Economic Development | S_4-1_ | 0.0167 | 0.0194 | 0.0249 | 0.0351 | 0.0444 | 0.0584 | 0.0652 | 0.0798 |
|  |  | S_4-2_ | 0.1263 | 0.1255 | 0.1269 | 0.1198 | 0.1287 | 0.1274 | 0.1254 | 0.1279 |
|  |  | S_4-3_ | 0.1393 | 0.1373 | 0.1380 | 0.1297 | 0.1381 | 0.1352 | 0.1312 | 0.1325 |

Table. S20. Chongzuo obstacle degree analysis data.

| **System** | **Criteria layer** | **Indicators (units)** | **2019** | **2018** | **2017** | **2016** | **2015** | **2014** | **2013** | **2012** |
| --- | --- | --- | --- | --- | --- | --- | --- | --- | --- | --- |
| Support | Climate Conditions | S_1-1_ | 0.0091 | 0.0090 | 0.0085 | 0.0086 | 0.0031 | 0.0007 | 0.0004 | 0.0033 |
|  |  | S_1-2_ | 0.0104 | 0.0109 | 0.0085 | 0.0090 | 0.0087 | 0.0136 | 0.0023 | 0.0063 |
|  |  | S_1-3_ | 0.0115 | 0.0122 | 0.0114 | 0.0082 | 0.0040 | 0.0021 | 0.0017 | 0.0042 |
|  |  | S_2-1_ | 0.0638 | 0.0668 | 0.0576 | 0.0589 | 0.0542 | 0.0439 | 0.0478 | 0.0562 |
|  |  | S_2-2_ | 0.0731 | 0.0724 | 0.0684 | 0.0559 | 0.0570 | 0.0554 | 0.0559 | 0.0554 |
|  | Resource Endowment | S_2-3_ | 0.0459 | 0.0457 | 0.0434 | 0.0356 | 0.0342 | 0.0325 | 0.0332 | 0.0340 |
|  |  | S_2-4_ | 0.1677 | 0.1661 | 0.1569 | 0.1282 | 0.1384 | 0.1372 | 0.1385 | 0.1344 |
|  |  | S_2-5_ | 0.0570 | 0.0564 | 0.0533 | 0.0819 | 0.0613 | 0.0890 | 0.0794 | 0.0658 |
|  |  | S_2-6_ | 0.0566 | 0.0561 | 0.0530 | 0.0525 | 0.0456 | 0.0342 | 0.0392 | 0.0396 |
|  | Environmental Governance | S_3-1_ | 0.0306 | 0.0286 | 0.0284 | 0.0280 | 0.0285 | 0.0259 | 0.0429 | 0.0522 |
|  |  | S_3-2_ | 0.0183 | 0.0126 | 0.0129 | 0.0589 | 0.0550 | 0.0522 | 0.0441 | 0.0439 |
|  |  | S_3-3_ | 0.0094 | 0.0090 | 0.0085 | 0.0543 | 0.0566 | 0.0609 | 0.0561 | 0.0522 |
|  |  | S_3-4_ | 0.1905 | 0.1892 | 0.1771 | 0.1438 | 0.1538 | 0.1549 | 0.1552 | 0.1525 |
|  | Economic Development | S_4-1_ | 0.0091 | 0.0184 | 0.0721 | 0.0736 | 0.0801 | 0.0793 | 0.0808 | 0.0810 |
|  |  | S_4-2_ | 0.0932 | 0.0946 | 0.0961 | 0.0854 | 0.0934 | 0.0932 | 0.0965 | 0.0963 |
|  |  | S_4-3_ | 0.1535 | 0.1520 | 0.1436 | 0.1173 | 0.1261 | 0.1249 | 0.1261 | 0.1226 |

# 10. Mine RECC Index in 2012-2019.

Table. S21. Mine RECC Index in 2012-2019.

| **county** | **years** | **Pressure index** | **Support index** | **RECC index** | **county** | **years** | **Pressure index** | **Support index** | **RECC index** |
| --- | --- | --- | --- | --- | --- | --- | --- | --- | --- |
| Bayan Obo | 2019 | 0.5253 | 0.4188 | 1.2543 | Mianning | 2019 | 0.2596 | 0.4073 | 0.6373 |
|  | 2018 | 0.5139 | 0.4343 | 1.1833 |  | 2018 | 0.2622 | 0.4264 | 0.6149 |
|  | 2017 | 0.5151 | 0.4105 | 1.2547 |  | 2017 | 0.2632 | 0.4044 | 0.6510 |
|  | 2016 | 0.5239 | 0.4191 | 1.2500 |  | 2016 | 0.2651 | 0.4099 | 0.6468 |
|  | 2015 | 0.6262 | 0.4926 | 1.2711 |  | 2015 | 0.2816 | 0.4731 | 0.5952 |
|  | 2014 | 0.5335 | 0.4927 | 1.0827 |  | 2014 | 0.2828 | 0.4704 | 0.6011 |
|  | 2013 | 0.4895 | 0.4767 | 1.0268 |  | 2013 | 0.2844 | 0.4553 | 0.6246 |
|  | 2012 | 0.3382 | 0.4510 | 0.7498 |  | 2012 | 0.2821 | 0.4451 | 0.6338 |
| Weishan | 2019 | 0.0993 | 0.3112 | 0.3191 | Pingyuan | 2019 | 0.1545 | 0.4247 | 0.3637 |
|  | 2018 | 0.1011 | 0.3287 | 0.3077 |  | 2018 | 0.1540 | 0.4162 | 0.3699 |
|  | 2017 | 0.1069 | 0.2997 | 0.3566 |  | 2017 | 0.1552 | 0.4195 | 0.3699 |
|  | 2016 | 0.1084 | 0.2973 | 0.3647 |  | 2016 | 0.1577 | 0.3823 | 0.4124 |
|  | 2015 | 0.1099 | 0.3593 | 0.3059 |  | 2015 | 0.1606 | 0.4471 | 0.3591 |
|  | 2014 | 0.1113 | 0.3648 | 0.3052 |  | 2014 | 0.1577 | 0.4446 | 0.3546 |
|  | 2013 | 0.1016 | 0.3553 | 0.2859 |  | 2013 | 0.1653 | 0.4278 | 0.3864 |
|  | 2012 | 0.0910 | 0.3241 | 0.2808 |  | 2012 | 0.1439 | 0.4241 | 0.3393 |
| Longnan | 2019 | 0.4281 | 0.5056 | 0.8467 | Chongzuo | 2019 | 0.0384 | 0.4468 | 0.0860 |
|  | 2018 | 0.4276 | 0.5004 | 0.8545 |  | 2018 | 0.0398 | 0.4414 | 0.0902 |
|  | 2017 | 0.4290 | 0.4776 | 0.8983 |  | 2017 | 0.0388 | 0.4087 | 0.0949 |
|  | 2016 | 0.4290 | 0.4657 | 0.9212 |  | 2016 | 0.0472 | 0.2763 | 0.1709 |
|  | 2015 | 0.3944 | 0.5193 | 0.7595 |  | 2015 | 0.0324 | 0.3568 | 0.0908 |
|  | 2014 | 0.4297 | 0.5040 | 0.8526 |  | 2014 | 0.0416 | 0.3608 | 0.1154 |
|  | 2013 | 0.4287 | 0.4636 | 0.9248 |  | 2013 | 0.0368 | 0.3665 | 0.1003 |
|  | 2012 | 0.2729 | 0.4894 | 0.5575 |  | 2012 | 0.0424 | 0.3381 | 0.1253 |

# References

1. Wang, J. M.; Wang, P.; Qin, Q.; Wang, H. D., The effects of land subsidence and rehabilitation on soil hydraulic properties in a mining area in the Loess Plateau of China. Catena 2017, 159, 51-59.

2. Halwatura, D.; Lechner, A. M.; Arnold, S., Drought severity-duration-frequency curves: a foundation for risk assessment and planning tool for ecosystem establishment in post-mining landscapes. Hydrol. Earth Syst. Sci. 2015, 19 (2), 1069-1091.

3. Gajic, G.; Djurdjevic, L.; Kostic, O.; Jaric, S.; Mitrovic, M.; Pavlovic, P., Ecological Potential of Plants for Phytoremediation and Ecorestoration of Fly Ash Deposits and Mine Wastes. Front. Environ. Sci. 2018, 6, 24.

4. Vickers, H.; Gillespie, M.; Gravina, A., Assessing the development of rehabilitated grasslands on post-mined landforms in north west Queensland, Australia. Agriculture Ecosystems & Environment 2012, 163 (none), 72-84.

5. Lingyan; Zhou; Zhaolong; Li; Wen; Liu; Shenghong; Liu; Limin; Zhang, Restoration of rare earth mine areas: organic amendments and phytoremediation. Environmental Science&Pollution Research 2015.

6. Wasylycia-Leis, J.; Fitzpatrick, P.; Fonseca, A., Mining Communities from a Resilience Perspective: Managing Disturbance and Vulnerability in Itabira, Brazil. Environmental Management 2014, 53 (3), 481-495.

7. Zhang, J.; Fu, M.; Tao, J.; Huang, Y.; Hassani, F. P.; Bai, Z., Response of ecological storage and conservation to land use transformation: A case study of a mining town in China. Ecological Modelling 2010, 221 (10), 1427-1439.

8. Koch; John; M.; Gove; Aaron; D.; Didham; Raphael; K.; Grigg, Long-term data suggest jarrah-forest establishment at restored mine sites is resistant to climate variability. The Journal of Ecology 2015, 103 (1), 78-89.

9. Wu, X. A.; Xlb, C.; Yzb, C.; Hsb, C.; Jlb, C., Ecological resilience assessment of an arid coal mining area using index of entropy and linear weighted analysis: A case study of Shendong Coalfield, China - ScienceDirect. Ecological Indicators 109.

10. Mohapatra, H.; Goswami, S., Assessment and analysis of noise levels in and around Ib river coalfield, Orissa, India. Journal of Environmental Biology 2012, 33 (3), 649.

11. Wu, Z.; Lei, S.; He, B. J.; Bian, Z.; Wang, Y.; Lu, Q.; Peng, S.; Duo, L., Assessment of Landscape Ecological Health: A Case Study of a Mining City in a Semi-Arid Steppe. International Journal of Environmental Research and Public Health 2019, 16 (5).

12. Tai, X.; Xiao, W.; Tang, Y., A quantitative assessment of vulnerability using social-economic-natural compound ecosystem framework in coal mining cities. Journal of Cleaner Production 2020, 120969.

13. Liu, S. L.; Li, W. P., Zoning and management of phreatic water resource conservation impacted by underground coal mining: A case study in arid and semiarid areas. Journal of Cleaner Production 2019, 224, 677-685.

14. Sozen, S.; Orhon, D.; Dincer, H.; Atesok, G.; Basturkcu, H.; Yalcm, T.; Oznesil, H.; Karaca, C.; Alli, B.; Dulkadiroglu, H., Resource recovery as a sustainable perspective for the remediation of mining wastes: rehabilitation of the CMC mining waste site in Northern Cyprus. Bulletin of Engineering Geology and the Environment 2017, 76 (4), 1535-1547.

15. Martins, W.; Lima, M.; Junior, U.; Amorim, L.; Schwartz, G., Ecological methods and indicators for recovering and monitoring ecosystems after mining: A global literature review. Ecological Engineering 2020, 145.

16. Yang, Y.; Li, Y.; Chen, F.; Zhang, S.; Hou, H., Regime shift and redevelopment of a mining area's socio-ecological system under resilience thinking: a case study in Shanxi Province, China. Environment, Development and Sustainability: A Multidisciplinary Approach to the Theory and Practice of Sustainable Development 2018.

17. Thorne, M.; Cardina, J., Prairie Grass Establishment on Calcareous Reclaimed Mine Soil. Journal of Environmental Quality 2011, 40 (6).

18. Ahirwal, J.; Pandey, V. C., Restoration of mine egraded land for sustainable environmental development. Restoration Ecology 2020.

19. Niu, F.; Yang, X.; Zhang, X., Application of an evaluation method of resource and environment carrying capacity in the adjustment of industrial structure in Tibet. 2020.

20. Wang, X.; Tan, K.; Xu, K.; Chen, Y.; Ding, J., Quantitative Evaluation of the Eco-Environment in a Coalfield Based on Multi-Temporal Remote Sensing Imagery: A Case Study of Yuxian, China. International Journal of Environmental Research & Public Health 2019, 16 (3).

21. Liu, L.; Zhou, J. S., Analyzing Ecological Functions in Coal Mining Cities Based on RS and GIS. Pol. J. Environ. Stud. 2018, 27 (3), 1165-1174.

22. Wu, X.; Zhang, J.; Geng, X.; Wang, T.; Liu, S., Increasing green infrastructure-based ecological resilience in urban systems: A perspective from locating ecological and disturbance sources in a resource-based city. Sustainable Cities and Society 2020, 61, 102354.

23. Cardoso, E. B.; Júnior, P.; Silva, M.; Cerqueira, A.; Jordo, T. C.; Moreira, B. C.; Pereira, E. G.; Kasuya, M., Composition and diversity of prokaryotes at an iron ore post‐mining site revealed the natural resilience 10years after mining exploitation. Land Degradation & Development.

24. Cao, D. Y.; Huang, C. L.; Wu, J.; Li, H. T.; Zhang, Y. D., Environment Carrying Capacity Evaluation of Coal Mining in Shanxi Province. Advanced Materials Research 2012, 518-523, 1141-1144.

25. Chi, M.; Zhang, D.; Zhao, Q.; Yu, W.; Liang, S., Determining the scale of coal mining in an ecologically fragile mining area under the constraint of water resources carrying capacity. Journal of Environmental Management 2020, 279 (2), 111621.

26. Matlaba, V. J.; Pereira, L. R.; Mota, J. A.; Santos, J., Resilience Perception of a Mining Town in Eastern Amazonia: A Case Study of Cana Dos Carajás, Brazil. Environmental Management 2021, 1-19.

27. Zhu, X.; Zhou, Y.; Yang, Y.; Hou, H.; Liu, R., Estimation of the Restored Forest Spatial Structure in Semi-Arid Mine Dumps Using Worldview-2 Imagery. Forests 2020, 11 (6), 695.

28. Yang, H.; Zhai, G.; Zhang, Y., Ecological vulnerability assessment and spatial pattern optimization of resource-based cities: A case study of Huaibei City, China. Human and Ecological Risk Assessment 2020, (11), 1-20.

29. Harahap, F. R.; Taqwa, R.; Juniah, R.; Wildayana, E., Sustainability for Management and Protection Tin Mining Environment. In 1st Sriwijaya International Conference on Environmental Issues 2018, Amin, M., Ed. E D P Sciences: Cedex A, 2018; Vol. 68.

30. Zhang, G. W.; Wang, L. J.; Fang, J.; Ca O, X. K., Choice and Application of Governance Mode of Mining Subsidence Areas in Cities. Advanced Materials Research 2012, 524-527, 111-117.

31. Wang, X.; Zhang, J., Measurement on Environmental Carrying Capacity of Coal Industry: Shaanxi as an Example. 2010; p 402-407.

32. Introduction, T. M. E. w. a.; Gilbert, N. b. G., An essay on the principle of population. An essay on the principle of population ;: 1878.

33. Jin, Y.; Jin, X.; Chen, L. I., Applying supporting-pressuring coupling curve to the evaluation of urban land carrying capacity:The case study of 32 cities in Zhejiang province. Geographical Research 2018.

34. Hadwen, S.; Palmer, L. J., Reindeer in Alaska. 1922.

35. Holling, Resilience and Stability of Ecological Systems. Annual Review of Ecology and Systematics 1973.

36. Munasinghe, M., Making economic growth more sustainable. Ecological Economics 1995, 15 (2), 121-124.

37. Chapman; EJ; Byron; CJ, The flexible application of carrying capacity in ecology. Glob Ecol Conserv 2018.

38. Sun; Chuanwang; Chen; Litai; Tian; Yuan, Study on the urban state carrying capacity for unbalanced sustainable development regions: Evidence from the Yangtze River Economic Belt. Ecological Indicators Integrating Monitoring Assessment & Management 2018.

39. Ma, P.; Ye, G.; Peng, X.; Liu, J.; Qi, J.; Jia, S., Development of an index system for evaluation of ecological carrying capacity of marine ecosystems. Ocean & Coastal Management 2017, 144 (jul.), 23-30.

40. Zhang, F.; Wang, Y.; Ma, X.; Wang, Y.; Yang, G.; Zhu, L., Evaluation of resources and environmental carrying capacity of 36 large cities in China based on a support-pressure coupling mechanism. The Science of the Total Environment 2019, 688 (Oct.20), 838-854.

41. Wei, X.; Shen, L.; Liu, Z.; Luo, L.; Chen, Y., Comparative analysis on the evolution of ecological carrying capacity between provinces during urbanization process in China. Ecological Indicators 2020, 112, 106179.

42. Wu, X.; Hu, F., Analysis of ecological carrying capacity using a fuzzy comprehensive evaluation method. Ecological Indicators 2020, 113, 106243-.

43. Wang; Jiayang; Wei; Xiaomei; Guo; Qian, A three-dimensional evaluation model for regional carrying capacity of ecological environment to social economic development: Model development and a case study in China. Ecological Indicators Integrating Monitoring Assessment & Management 2018.

44. Jia, Z.; Cai, Y.; Chen, Y.; Zeng, W., Regionalization of water environmental carrying capacity for supporting the sustainable water resources management and development in China. Resources, Conservation and Recycling 2018, 134, 282-293.

45. Mza, B.; Lsa, B.; Vwyt, C.; Zhi, L.; Tsa, B.; Wla, B., A load-carrier perspective examination on the change of ecological environment carrying capacity during urbanization process in China. Science of The Total Environment 714.

46. Tw, A.; Shan, S. A.; Sw, B.; Yy, A.; Ml, A., Remote sensing assessment and spatiotemporal variations analysis of ecological carrying capacity in the Aral Sea Basin - ScienceDirect. Science of The Total Environment 735.

47. Peng, B.; Li, Y.; Elahi, E.; Wei, G., Dynamic evolution of ecological carrying capacity based on the ecological footprint theory: A case study of Jiangsu province. Ecological indicators 2019, 99 (APR.), 19-26.

48. Wu, L.; Su, X.; Ma, X.; Kang, Y.; Jiang, Y., Integrated modeling framework for evaluating and predicting the water resources carrying capacity in a continental river basin of Northwest China. Journal of Cleaner Production 2018, 204 (PT.1-1178), 366-379.

49. Feng, H.; Xie, S. Y.; Liu, F.; Cui, Y.; Chen, C., Evaluation of Resources and Environment Carrying Capacity Based on Topsis of Grey Correlation Method and GIS in Chongqing City. Journal of Southwest University (Natural Science Edition) 2017.

50. Irankhahi, M.; Jozi, S. A.; Farshchi, P.; Shariat, S. M.; Liaghati, H., Combination of GISFM and TOPSIS to evaluation of Urban Environment Carrying Capacity (Case study: Shemiran City, Iran). International journal of Environmental Science and Technology 2017, 14 (6), 1-16.

51. A, Y. C.; A, S. Z.; A, Y. Z.; A, L. X.; A, Z. Q.; A, G. S.; B, J. Z., Comprehensive assessment and hierarchical management of the sustainable utilization of urban water resources based on catastrophe theory - ScienceDirect. Journal of the Taiwan Institute of Chemical Engineers 2016, 60, 430-437.

52. Fan, X. G.; Mi, W. B.; Ma, Z. N., [Construction and application of economy-pollution-environment three-dimensional evaluation model for district]. Huan jing ke xue= Huanjing kexue / [bian ji, Zhongguo ke xue yuan huan jing ke xue wei yuan hui "Huan jing ke xue" bian ji wei yuan hui.] 2015, 36 (2), 751-758.

53. Ge, W.; Cxabc, D.; Zqabc, D.; Fmabc, D.; Xlabc, D., Development tendency analysis for the water resource carrying capacity based on system dynamics model and the improved fuzzy comprehensive evaluation method in the Changchun city, China - ScienceDirect. Ecological Indicators 122.

54. Tian, Y.; Sun, C., Comprehensive Carrying Capacity, Economic Growth and the Sustainable Development of Urban Areas: A Case Study of the Yangtze River Economic Belt. Journal of Cleaner Production 2018, 195 (SEP.10), 486-496.

55. Han, B.; Liu, H.; Wang, R., Urban ecological security assessment for cities in the Beijing–Tianjin–Hebei metropolitan region based on fuzzy and entropy methods. Ecological Modelling 2015, 318 (1), 217-225.

56. Feng, H.; Cgo, B.; Xi, C.; Qc, A.; Dz, A., An entropy-based investigation into the impact of ecological water diversion on land cover complexity of restored oasis in arid inland river basins. Ecological Engineering 151.

57. Peng, W.; Wang, X.; Li, X.; He, C., Sustainability evaluation based on the emergy ecological footprint method: A case study of Qingdao, China, from 2004 to 2014. Ecological Indicators 2018, 85 (FEB.), 1249–1261.

58. Chen, Y.; Lu, H.; Yan, P.; Yang, Y.; Xia, J., Analysis of water–carbon–ecological footprints and resource–environment pressure in the Triangle of Central China. Ecological Indicators 2021, 125 (7401), 107448.

59. Hp, A.; Mz, A.; Yong, G.; Fei, W. A.; Hd, A., Emergy-based ecological footprint analysis for a mega-city: The dynamic changes of Shanghai. Journal of Cleaner Production 2019, 210, 552-562.

60. Okey, T. A., Indicators of marine ecosystem integrity for Canada's Pacific: An expert-based hierarchical approach. Science of The Total Environment 2018, 645 (DEC.15), 1114-1125.

61. Zeng, C.; Liu, Y.; Liu, Y.; Hu, J.; Bai, X.; Yang, X., An Integrated Approach for Assessing Aquatic Ecological Carrying Capacity: A Case Study of Wujin District in the Tai Lake Basin, China. International Journal of Environmental Research and Public Health 2011, 8 (1).

62. Ying, L.; Guo, T.; Jing, Z., Research of Ecological Carrying Capacity ---Comprehensive Evaluation Model. Procedia Environmental Sciences 2011, 11 (part-PB), 864-868.

63. Li, X., TOPSIS Model with Entropy Weight for Eco Geological Environmental Carrying Capacity Assessment. Microprocessors and Microsystems 2021, 103805.

64. Xu, X.; Zhang, Z.; Long, T.; Sun, S.; Gao, J., Mega-city region sustainability assessment and obstacles identification with GIS–Entropy–TOPSIS model: A case in Yangtze River Delta urban Agglomeration, China. Journal of Cleaner Production 2021, 294, 126147.

65. Liu, E. N.; Wang, Y.; Chen, W.; Chen, W.; Ning, S., Evaluating the transformation of China's resource-based cities: An integrated sequential weight and TOPSIS approach. Socio-Economic Planning Sciences 2021, (17), 101022.

66 Balaram, V. Rare earth elements: A review of applications, occurrence, exploration, analysis, recycling, and environmental impact. Geosci Front 10(4), 1285-1303(2019).

67 Wang Z, et al. Characteristics and Evaluation of Soil Rare Earth Element Pollution in the Bayan Obo Mining Region of Inner Mongolia. Huanjing kexue 42(3), 1503-1513 (2021).

68 Lompré J S, et al. Bioaccumulation and ecotoxicological responses of clams exposed to terbium and carbon nanotubes: Comparison between native (Ruditapes decussatus) and invasive (Ruditapes philippinarum) species. Sci Total Environ 784, 146914 (2021).

69 Trapasso, G., Chiesa, S., Freitas, R., & Pereira, E. What do we know about the ecotoxicological implications of the rare earth element gadolinium in aquatic ecosystems? Sci Total Environ 781, 146273 (2021).

70 Yin, X., Martineau, C., Demers, I., Basiliko, N., & Fenton, N. J. The potential environmental risks associated with the development of rare earth element production in Canada. Environ Rev 4, 354-377 (2021).

71 Barakos G, Mischo H, Gutzmer J. Rare earth underground mining approaches with respect to radioactivity control and monitoring strategies, Rare Earths Industry 121-138, (2016).

72 Zheng, C. L. et al. Rare Earth Distribution in the Soil around Rare Earth Tailings. Chinese Rare Earths 37, 73-80 (2016).

73 Feng, X., Li, H. K. & Li, Y. S. Ecological environment quality evaluation and evolution analysis of a rare earth mining area under different disturbance conditions. Environ. Geochem. Health 43, 2243-2256 (2021).

74 Liu W. S, et al. Water, sediment and agricultural soil contamination from an ion-adsorption rare earth mining area. Chemosphere 216, 75-83 (2019).

75 Emmanuel, E. C., Ananthi, T., Anandkumar, B., & Maruthamuthu, S. Accumulation of rare earth elements by siderophore-forming Arthrobacter luteolus isolated from rare earth environment of Chavara, India. J. Biosci. 37, 25-31, (2012).

76 Rodrigues E S, et al. Effect of nano cerium oxide on soybean (Glycine max L. Merrill) crop exposed to environmentally relevant concentrations. Chemosphere 273, 128492 (2020).

77 Adeel M, et al. Bio-interaction of nano and bulk lanthanum and ytterbium oxides in soil system: Biochemical, genetic, and histopathological effects on Eisenia fetida. J Hazard Mater 415, 125574 (2021).

78 Trapasso G, et al. How Ulva lactuca can influence the impacts induced by the rare earth element Gadolinium in Mytilus galloprovincialis? The role of macroalgae in water safety towards marine wildlife. Ecotoxicol Environ Saf 215, 112101 (2021).

79 Liang, Z, et al. Soil characteristics and microbial community response in rare earth mining areas in southern Jiangxi Province, China. Environ Sci Pollut Res 28, 56418–56431 (2021)..

80 Jomaa M, Dieme D, Desrosiers M, et al. Effect of the dose on the toxicokinetics of a quaternary mixture of rare earth elements administered to rats. Toxicol Lett 345, 46-53 (2021).

81 He, X., Yuan, T., Jiang, X., Yang, H., & Zheng, C. L. Effects of contaminated surface water and groundwater from a rare earth mining area on the biology and the physiology of Sprague-Dawley rats.4. Sci Total Environ 761, 144123 (2021).

82 Singh, P. et al. Biotite as a geoindicator of rare earth element contamination in Gomati River Basin, Ganga Alluvial Plain, northern India. Environ Monit Assess 193(6), 361-361 (2021).

83 El Zrelli, R. et al. Rare earth elements characterization associated to the phosphate fertilizer plants of Gabes (Tunisia, Central Mediterranean Sea): Geochemical properties and behavior, related economic losses, and potential hazards. Sci Total Environ 791, 148268 (2021).

84 Shen, J. L. et al. Adsorption behavior and mechanism of Serratia marcescens for Eu(III) in rare earth wastewater. Environ Sci Pollut Res 28, 56915-56926 (2021).

85 Liu, H. Y. et al. Geochemical signatures of rare earth elements and yttrium exploited by acid solution mining around an ion-adsorption type deposit: Role of source control and potential for recovery. Sci Total Environ 804, 150241 (2022).

86 Sprecher, B. et al. Life Cycle Inventory of the Production of Rare Earths and the Subsequent Production of NdFeB Rare Earth Permanent Magnets. Environ Sci Technol 48(7), 3951 (2014).

87 Kramer, K. et al. Collaborative study to improve the quality control of rare earth element determinations in environmental matrices. J Environ Monit 1(1), 83-89 (1999).

88 Kramer, K. et al. Certified reference materials for the quality control of rare earth element determinations in the environment. Trends Analyt Chem 21(11), 762-773 (2002).

89 Saaty, T. L. Axiomatic foundation of the analytic hierarchy process. Manag Sci 32(7), 841-855 (1986).

90 Rubinstein, R. The Cross-Entropy Method for Combinatorial and Continuous Optimization. Methodol Comput Appl Probab 1, 127-190 (1999).
